# Approaches to *ab initio* molecular replacement of α-helical transmembrane proteins

**DOI:** 10.1107/S2059798317016436

**Published:** 2017-11-22

**Authors:** Jens M. H. Thomas, Felix Simkovic, Ronan Keegan, Olga Mayans, Chengxin Zhang, Yang Zhang, Daniel J. Rigden

**Affiliations:** a Institute of Integrative Biology, University of Liverpool, Liverpool L69 7ZB, England; b Research Complex at Harwell, STFC Rutherford Appleton Laboratory, Didcot OX11 0FA, England; c Fachbereich Biologie, Universität Konstanz, D-78457 Konstanz, Germany; d Department of Computational Medicine and Bioinformatics, Department of Biological Chemistry, Medical School, University of Michigan, 100 Washtenaw Avenue, Ann Arbor, MI 48109-2218, USA

**Keywords:** transmembrane proteins, *ab initio* phasing, *ab initio* modelling, predicted contacts

## Abstract

α-Helical transmembrane proteins are a ubiquitous and important class of proteins, but present difficulties for crystallographic structure solution. Here, the effectiveness of the *AMPLE* molecular replacement pipeline in solving α-helical transmembrane-protein structures is assessed using a small library of eight ideal helices, as well as search models derived from *ab initio* models generated both with and without evolutionary contact information. The ideal helices prove to be surprisingly effective at solving higher resolution structures, but *ab initio*-derived search models are able to solve structures that could not be solved with the ideal helices. The addition of evolutionary contact information results in a marked improvement in the modelling and makes additional solutions possible.

## Abstract 2

Homology-independent methods for *ab initio* phasing of α-helical transmembrane proteins are explored.

## 1. Introduction   

Transmembrane proteins are an important class of proteins that are estimated to comprise about 30% of the proteome (Tusnády *et al.*, 2004 ▸). They reside, at least partly and often predominantly, within the hydrophobic cell membrane, sandwiched between the aqueous cell interior and exterior. Transmembrane proteins come in two main forms, α-helical and β-barrel, with the overwhelming majority being of the α-helical form (White & Wimley, 1999 ▸). Estimates of the number of transmembrane proteins encoded in the human genome vary. Most studies agree that roughly 26% of proteins are transmembrane proteins, but this includes a large number of single-pass transmembrane proteins. Polytopic proteins, as studied here, are thought to represent around 14% of the human proteome (Almén *et al.*, 2009 ▸; Fagerberg *et al.*, 2010 ▸).

Conventional X-ray crystallography is still the predominant mode of structure solution for transmembrane proteins. Unfortunately, their hydrophobic nature means that transmembrane proteins are particularly challenging to work with experimentally. Detergents are usually required to extract the protein from the membrane environment. These surround the protein, mimicking the membrane environment and forming a water-soluble protein–detergent complex (PDC). The detergent coating of the PDC reduces the number of protein–protein contacts available to guide crystal formation, resulting in large fragile crystals with a high solvent content (Moraes *et al.*, 2014 ▸). The nature of these crystals means that they often diffract poorly and are unstable in an X-ray beam. As a result of these complications, of the more than 130 000 protein structures currently deposited in the PDB (Berman *et al.*, 2000 ▸), fewer than 3% (3084) are classified as transmembrane proteins by the *TMDET* algorithm (Tusnády *et al.*, 2004 ▸). The low number of structures means that the probability of finding homologous structures to use for molecular replacement (MR) can often be low, so that the use of unconventional approaches may be required. These can include methods based on fragments or libraries of fragments, such as that employed by *ARCIMBOLDO*, *ARCIMBOLDO_LITE* and *ARCIMBOLDO_BORGES* (Rodríguez *et al.*, 2009 ▸; Sammito *et al.*, 2013 ▸, 2015 ▸; Millán *et al.*, 2015 ▸), or those that employ *ab initio* structure prediction (Bibby *et al.*, 2012 ▸; Shrestha *et al.*, 2011 ▸; Shrestha & Zhang, 2015 ▸; Wang *et al.*, 2016 ▸).

*Ab initio* protein-structure prediction is the process of determining the tertiary structure of a protein starting purely from its sequence, and not relying on the use of an existing structure as a template. Popular examples of *ab initio* modelling software include *ROSETTA*, *QUARK* and *SAINT*2 (Simons *et al.*, 1997 ▸; Rohl *et al.*, 2004 ▸; Xu *et al.*, 2012 ▸; Ellis *et al.*, 2010 ▸). *Ab initio* modelling for globular proteins, however, is accurate only for modest chain lengths. For example, in the absence of additional information, reliable modelling with *ROSETTA* is still limited to sequences of up to 130 residues (Tai *et al.*, 2014 ▸), so that models of larger proteins are not usually sufficiently accurate to be suitable for MR.

Our program *AMPLE* uses a cluster-and-truncate procedure to extract ensembles for MR from the initial decoys generated by *ab initio* programs (Bibby *et al.*, 2012 ▸). *AMPLE* constructs MR search ensembles by generating 1000 *ab initio* models and then taking up to 200 models from the top *SPICKER* cluster (Zhang & Skolnick, 2004 ▸) and truncating them into 20 evenly-spaced size bins based on per-residue structural variance measurements. The truncated models are then subclustered under 1, 2 or 3 Å radius r.m.s.d. thresholds, before being subjected to three different side-chain treatments: polyalanine (removal of all side chains), ‘reliable’ (retaining side chains for residues with fewer rotamers) and all-atom (no editing). The search ensembles are subjected to MR with *Phaser* (McCoy *et al.*, 2007 ▸; Read & McCoy, 2016 ▸) and MR-positioned models are subjected to density modification and automated main-chain tracing using *SHELXE* (Thorn & Sheldrick, 2013 ▸). Further structure rebuilding of the *SHELXE* chain traces with *ARP*/*wARP* (Langer *et al.*, 2008 ▸) and/or *Buccaneer* (Cowtan, 2006 ▸) can also be undertaken.

Despite the usually poor overall quality of the models for larger proteins (which means that the individual models are unsuitable for MR), the *AMPLE* algorithm is able to generate successful ensembles for MR, and we have seen successes with targets of up to 250 residues in length for α-helical coiled-coil proteins (Thomas *et al.*, 2015 ▸) and up to 221 residues for cases where contact predictions (see below) were available to assist the modelling of gobular proteins (Simkovic *et al.*, 2016 ▸). The more regular and ordered nature of α-helices, as opposed to β-sheets or loops, facilitates *ab initio* modelling for structures containing these secondary-structure elements. In addition, the membrane-spanning helices of an α-helical transmembrane protein can be reliably predicted and assumed to remain within a layer of finite width. This helpfully limits the conformational space to be explored during the *ab initio* modelling, aiding model accuracy and potentially raising the upper size limit of tractable targets. Thus, *ab initio* modelling might be particularly suitable for transmembrane proteins (Yarov-Yarovoy *et al.*, 2006 ▸) and make the resulting predictions good candidates for solution with *AMPLE*.

The accuracy of *ab initio* modelling may also be improved by the incorporation of additional information, one source of which is evolutionary covariance. In recent years, there has been a step change in the accuracy of residue–residue contact predictions generated from sequence information alone (Simkovic, Ovchinnikov *et al.*, 2017 ▸). These methods infer which residues are in physical contact by looking at the evolutionary covariance signal within an alignment of a family of homologous sequences. We have previously demonstrated the benefits of using such predictions in addressing challenging globular domains with *AMPLE* (Simkovic *et al.*, 2016 ▸).

Contact-prediction algorithms can generally be divided into two distinct categories: evolutionary coupling analysis and supervised machine learning. The former derives contact predictions by detecting evolutionary covariance amongst homologous sequences, and various implementations attempt this by employing a cooperative statistical model. Examples of implementations that employ a pseudo-likelihood maximization model can be found in applications such as *GREMLIN* (Ovchinnikov *et al.*, 2014 ▸) and *CCMPRED* (Seemayer *et al.*, 2014 ▸). Other implementations include sparse covariance matrix-inversion models such as *PSICOV* (Seemayer *et al.*, 2014 ▸; Jones *et al.*, 2012 ▸) or mean-field direct coupling analysis models such as *EVFold–mfDCA* (Kaján *et al.*, 2014 ▸).

Supervised machine-learning algorithms are not as effective as most evolutionary coupling analysis algorithms; however, in recent studies combining the two has proven to be the most effective use of both. These meta-predictors have been developed to combine predictions from a number of different prediction methods. *MetaPSICOV* is one such meta-predictor that uses a neural network to combine predictions from, amongst others, the *PSICOV*, *mfDCA* and *CCMPRED* scores (Jones *et al.*, 2015 ▸). A related approach is *MEMBRAIN*, which is a contact predictor optimized for transmembrane proteins (Jones *et al.*, 2015 ▸; Xiao & Shen, 2015 ▸). *MEMBRAIN* uses a neural network to combine a covariance-based approach (*PSICOV*) with the combination of a number of maximum-likelihood approaches, each of which trains a statistical model using sequence-derived features such as residue position along the transmembrane helix or sequence separation of residues. Most recently, *NeBcon* was proposed to combine multiple contact predictors from both coevolution and machine-learning techniques through naïve Bayes classifier and neural network training, which shows an advantage over the best individual predictors (He *et al.*, 2017 ▸).

Here, we explore the ability of *AMPLE* to solve transmembrane-protein structures using MR search ensembles derived from *ab initio* models from a variety of sources. Attempts were made to solve structures using models derived from *ROSETTA*’s established *ROSETTA­MEMBRANE* protocol and the *QUARK* modelling protocol, both of which do not use contact information by default. Solution was then attempted with *ROSETTA* models created using contact information derived from *GREMLIN*, *CCMPRED*, *Meta­PSICOV* or *MEMBRAIN* to test whether the additional contact information improved the success rate. As in our earlier work (Thomas *et al.*, 2015 ▸), we also tried to solve the structures with a library of short, ideal α-helices ranging in size from five to 40 residues. These were chosen for comparison with the highly truncated but model-derived α-helix-rich search models of a similar size that *AMPLE* frequently generates. These latter, unlike the ideal helices, are ensembles and can contain bent or kinked helices as well as other irregular features. The comparison therefore illuminates the contribution of the modelling and ensembling to MR success.

## 2. Methods   

### 2.1. Test-set selection   

**Figure 1:**
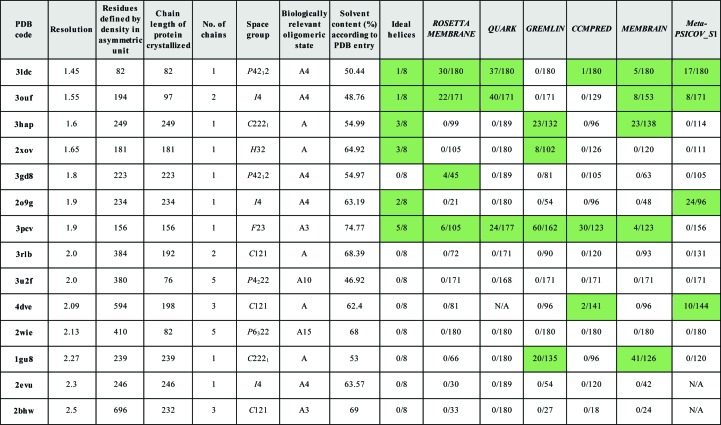
Selected data for the 14 transmembrane-protein targets with a summary of the MR solutions for each modelling protocol. For each modelling protocol, the number of successful search models over the total number of ensembles tested in *AMPLE* is shown, with cells with successful search models highlighted in green. The table is ordered by the resolution of the target.

A set of 14 transmembrane structures was selected by firstly generating a list of all the α-helical proteins from the PDBTM (Tusnády *et al.*, 2004 ▸). An advanced query was run against the PDB to extract all structures corresponding to the PDBTM list but containing a single protein entity (regardless of the number of copies in the crystallographic asymmetric unit), with a sequence length less than 250 and where the structure was resolved to a resolution of better than 2.5 Å. The structures were then clustered with *CD-HIT* (Li & Godzik, 2006 ▸) using a sequence-identity threshold of 0.4 and a word length of 2. For each cluster with more than one structure, the shortest, highest resolution structure was selected where there was a viable MTZ file [a binary file containing the reflection data from a crystallography experiment in *CCP*4 (Winn *et al.*, 2011 ▸) format]. Structures with a chain length of greater than 30 residues or with fewer than 800 residues in the asymmetric unit were selected to create the final set, as listed in Fig. 1 ▸, which also summarizes key data associated with each target and the results of running the different modelling protocols.

### 2.2. Ideal helices   

Following our work on coiled-coil proteins (Thomas *et al.*, 2015 ▸), *AMPLE* already contains a library of eight ideal polyalanine helices with residue lengths of five, ten, 15, 20, 25, 30, 35 and 40. The ideal helices were created using the *AVOGADRO* (Hanwell *et al.*, 2012 ▸) chemical editor and visualization application using a φ angle of −57.8° and a ψ angle of −47.0°.

### 2.3. *ROSETTA* modelling protocols   

The same set of *ROSETTA* fragments were used in the the *ROSETTAMEMBRANE*, *GREMLIN*, *CCMPRED*, *Meta­PSICOV* and *MEMBRAIN* protocols to facilitate comparison between them. The fragments were generated using the *ROSETTA* make_fragments.pl script supplying the -nohoms flag to ensure that no homologous fragments were used and so that no structural information from homologous structures was included in any modelling.

### 2.4. *ROSETTAMEMBRANE* modelling protocol   

The original *ROSETTAMEMBRANE* protocol (Yarov-Yarovoy *et al.*, 2006 ▸) was used. Yarov-Yarovoy and coworkers recommend only using *SAM* (*Sequence Alignment and Modeling System*; Katzman *et al.*, 2008 ▸) to predict the secondary structure, as the default *JUFO* (Meiler & Baker, 2003 ▸) and *PSIPRED* (Jones, 1999 ▸) predictors often incorrectly predict the secondary structure for transmembrane proteins. However, the recommended method for *AMPLE* users to generate the fragment database required by *ROSETTA* is to use the online *ROBETTA* server (Kim *et al.*, 2004 ▸), which does not support the use of *SAM*. Standard *ROSETTA* fragments, obtained using the default secondary-structure prediction protocol as used by the *ROBETTA* server, were therefore used as this replicates the procedure that will be followed by *AMPLE* users. In addition, this allowed the use of the same fragment databases with all the modelling protocols, as described above.

The *OCTOPUS* server (July 2015; Viklund & Elofsson, 2008 ▸) was used to predict transmembrane regions and a lipophilicity prediction was generated using the run_lips.pl script, which undertakes a *PSI-BLAST* (Altschul *et al.*, 1997 ▸) multiple sequence alignment of the target sequence against the NR database (O’Leary *et al.*, 2016 ▸). The *OCTOPUS* and lipophilicity predictions were then used with the *ROSETTAMEMBRANE* executable to generate the models. *ROSETTA* v.2015.22.57859 was used for the *ROSETTAMEMBRANE* modelling and all subsequent *ROSETTA* modelling in this work.

### 2.5. *QUARK* modelling   

The online *QUARK* server is a particularly easy way for *AMPLE* users to generate *ab initio* models, as it does not require them to install any additional software or use their local machine for the time-consuming model-generation stage (Keegan *et al.*, 2015 ▸). Although the *QUARK* modelling protocol (Xu *et al.*, 2012 ▸) does not feature any membrane-specific protocols, previous work by the developers has demonstrated success in modelling transmembrane proteins in *Escherichia coli* (Xu & Zhang, 2013 ▸). The *QUARK* decoys were generated on 20 November 2015. For each target, ten independent replica-exchange Monte Carlo (REMC) simulations were performed by *QUARK*, where each REMC runs 40 replicas in parallel at different temperatures with each replica containing 500 Monte Carlo cycles of simulations. This process output 50 000 decoys from the ten lowest-temperature replicas (*i.e.* ten REMC simulations × ten lowest-temperature replicas × 500 Monte Carlo cycles). From the full decoy set, a subset of 5000 decoys was randomly selected for further consideration. To reconstruct the full-atomic model from each of the selected C^α^ decoy structures, an initial all-atom representation was generated using *PULCHRA* 3.06 (Rotkiewicz & Skolnick, 2008 ▸) or *MaxSprout* 2006.10 (Holm & Sander, 1991 ▸) if *PULCHRA* was unsuccessful. Side chains were then added using *SCWRL* 4.0 (Krivov *et al.*, 2009 ▸).

In order to ensure that no homologous fragments were used in the modelling, PDB structures with a sequence identity of >30% to the target or that were detectable by *PSI-BLAST* (a criterion used by earlier *ab initio* folding benchmark tests; Simons *et al.*, 2001 ▸; Zhang *et al.*, 2003 ▸) were excluded from the *QUARK* fragment library.

### 2.6. *ROSETTA* modelling with *GREMLIN* predicted contacts   

The transmembrane-modelling protocol for modelling transmembrane proteins using contact predictions generated by the *GREMLIN* server (http://gremlin.bakerlab.org/) was developed by Ovchinnikov *et al.* (2015 ▸). Contact predictions generated by the *GREMLIN* server in November 2015 were used to generate the models. This method presents an attractive alternative to the original *ROSETTAMEMBRANE* protocol, as it only requires a user to generate the contact information using the *GREMLIN* server and then run the standard *ROSETTA* executables. There is no need to perform *OCTOPUS* transmembrane prediction or the lipophilicity prediction, with the associated need for *BLAST* and the NR database to be installed, as there is with *ROSETTA­MEMBRANE*. The protocol is described further in §S1.

### 2.7. Contact modelling (excluding *GREMLIN*)   

For each target, a multiple sequence alignment (MSA) was generated using the conkit-predict script from the *CONKIT* package (Simkovic, Thomas *et al.*, 2017 ▸) and using *HHblits* v.2.0.16 (Alva *et al.*, 2016 ▸) against UniProt20 v.2016_02 (The UniProt Consortium, 2017 ▸). A contact meta-prediction using *MetaPSICOV* v.1.04 (Jones *et al.*, 2015 ▸) was generated, which in turn used the following contact-prediction pipelines: *CCMPRED* v.0.3.2 (Seemayer *et al.*, 2014 ▸), *FreeContact* v.1.0.21 (Kaján *et al.*, 2014 ▸) and *PSICOV* v.2.1b3 (Jones *et al.*, 2012 ▸). The predictions from *MetaPSICOV* stage 1 (*MetaPSICOV_S*1) were used as the contact predictions for *MetaPSICOV* as recommended in Jones *et al.* (2015 ▸). The *CCMPRED* predictions generated by *MetaPSICOV* were used as the *CCMPRED* predictions. A set of contacts was also generated using the *MEMBRAIN* server v.2015-03-15.

For each set of contact predictions, the top *L* contact pairs were then turned into a set of restraints for *ROSETTA*, where *L* is the number of residues in the target sequence. The FADE energy function was employed with parameters identical to those reported in Michel *et al.* (2014 ▸). Modelling was then carried out with the *ROSETTA ABINITIO* protocol.

### 2.8. Molecular replacement   

All of the models were run in the automated MR pipeline *AMPLE* v.1.0 using default parameters (Bibby *et al.*, 2012 ▸) and with the *CCP*4 suite v.6.5.13 (Winn *et al.*, 2011 ▸), *SHELXE* v.2014/14 and *ARP*/*wARP* v.7.5.

To be considered a successful MR solution, the positioned models were required to yield a *SHELXE* correlation coefficient (CC) of at least 25.00 and an average chain length (ACL) of greater than 10.00. We imposed as an additional condition of success that structure rebuilding of the *SHELXE* chain traces with *ARP*/*wARP* (Langer *et al.*, 2008 ▸) and/or *Buccaneer* (Cowtan, 2006 ▸) resulted in an *R*_free_ value of 0.45 or better. A further validation was provided by measuring the weighted mean phase error between each rebuilt model and the crystal structure.

## 3. Results   

The weighted mean phase error for all of the final rebuilt models was measured to confirm that the structure had been correctly determined. In all cases the error was less than 30°.

### 3.1. Ideal helices   

Attempting solution with the *AMPLE* library of ideal helices solved six of the targets, as shown in Figs. 1 ▸ and 2 ▸.

The library of ideal helices was able to solve six targets in total. All targets smaller than 261 residues and with a resolution better than 1.9 Å were solved using this protocol, with the exception of target 3gd8. Considering that transmembrane proteins are considered to be relatively hard targets to solve, that so many of this set can be solved using a small library of ideal helices and a simple MR protocol is an encouraging result to set alongside other advances in the use of ideal helices (Millán *et al.*, 2015 ▸).

### 3.2. *ROSETTAMEMBRANE*   

Solution was then attempted using the *ROSETTA­MEMBRANE* protocol. Fig. 1 ▸ shows that *ROSETTA­MEMBRANE* performs more poorly than the *AMPLE* library of ideal helices, solving four targets. Three of the solved targets could be solved with the ideal helices. The single exception is 3gd8, which could now be solved for the first time. Targets 3hap, 2xov and 2o9g were not solved, despite being solvable with the ideal helices.

An analysis of the quality of the models was then made to determine how the effectiveness of the modelling affected the ability of *AMPLE* to solve the targets using *ROSETTAMEMBRANE*. The TM score of the complete model that became the centroid of the search ensemble was used as a metric for the quality of the models within the ensemble. A TM score of greater than 0.5 is generally considered to indicate correct prediction of the overall fold. The results of the analysis are displayed in Supplementary Fig. S2. The results show that there was no correlation between the quality of the models and the success of the ensembles, with the successful search models all coming from ensembles where the TM score of the complete centroid model was between 0.21 and 0.33. Targets 3u2f and 2wie failed to solve, despite some ensembles being derived from models with TM scores of 0.739 and 0.715, respectively.

**Figure 3:**
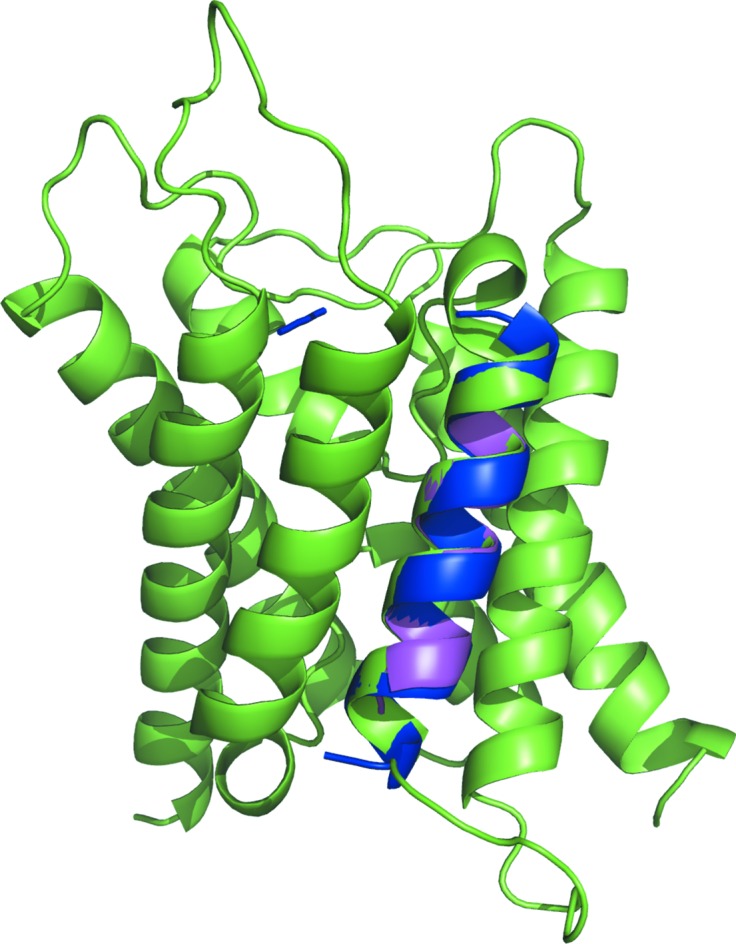
Successful solutions from ensembles c1_tl11_r2_allatom and c1_tl6_r3_reliable (blue and magenta, respectively) overlaid on the crystal structure of PDB entry 3gd8 (green).

Target 3gd8 could be solved with four ensembles, despite none of the successful ensembles being derived from a model with a TM score of better than 0.227. Selected data for the ensembles are listed in Supplementary Table S1. The RIO score (Thomas *et al.*, 2015 ▸), which assesses the in-sequence and out-of-sequence register overlap of the placed search-model residues (fragments of at least three residues) with the corresponding crystal structure, was zero for all solutions, indicating that the helices were not placed correctly with regard to sequence. Although the successful ensembles (c1_t11_r3_polyAla, c1_t11_r2_allatom, c1_t6_r3_reliable and c1_t6_r2_allatom) had undergone different subcluster radii and side-chain treatment, the final models for the successful search models c1_t11_r3_polyAla and c1_t11_r2_allatom were almost identical, as were those for c1_t6_r3_reliable and c1_t6_r2_allatom. [Ensembles in *AMPLE* are named using a quartet of identifiers separated by underscores. The first identifier is the number of the *SPICKER* cluster that the models were derived from, prefixed with a c, the second the truncation level, prefixed with a t, the third the subcluster radius, prefixed with an r, and the last the side-chain mode]. Fig. 3 ▸ shows the first of each pair (c1_t11_r2_allatom and c1_t6_r3_reliable) overlaid on the crystal structure.

The two solutions appeared to be largely straight helices of lengths of 25 and 14 residues, respectively. It is therefore interesting that the ideal helices of lengths 25 and 15 were unable to solve target 3gdb. An analysis of the placement of the helical segment in the two solutions of 3gd8 with *HELANAL* (Kumar & Bansal, 2012 ▸) identified the search models as being ‘curved’ and the helix of 3gd8 as being ‘kinked’ (Bansal *et al.*, 2000 ▸). A ‘kink’ is defined by the authors when the bending angle of a given residue is greater than 20° but less than 60°. The search ensembles generated by *AMPLE* contained between 19 and 30 search models, so it appears that the approximation of the slightly kinked helix by the slightly curved search model and/or diversity within the search-model ensembles is what enabled the *AMPLE* search ensemble to succeed where the *AMPLE* set of ideal helices failed. The *HELANAL* analysis and models of the search ensembles are displayed in Supplementary Table S2 and Supplementary Fig. S3, respectively.

### 3.3. *QUARK*   

Solution was then attempted with *QUARK* models. The results are displayed in Fig. 1 ▸, with a graphical summary in Supplementary Fig. S4 and an analysis of the TM scores in Supplementary Fig. S5. *QUARK* was able to solve three targets, all of which could be solved with the *AMPLE* library of ideal helices. An analysis of the TM scores for the models similar to that undertaken for *ROSETTAMEMBRANE* showed that the quality of the models again had little effect on solution. The *QUARK* models for target 3u2f were of even better quality than those for *ROSETTAMEMBRANE* (maximum TM score of 0.792), but the target could still not be solved. Overall, the quality of the *QUARK* models was rather better than for *ROSETTAMEMBRANE* (median TM score across all models of 0.385 as opposed to 0.263), but as the general quality of the modelling is poor, even the better models rarely seem to have the potential to generate solutions.

### 3.4. *GREMLIN*   

**Figure 4:**
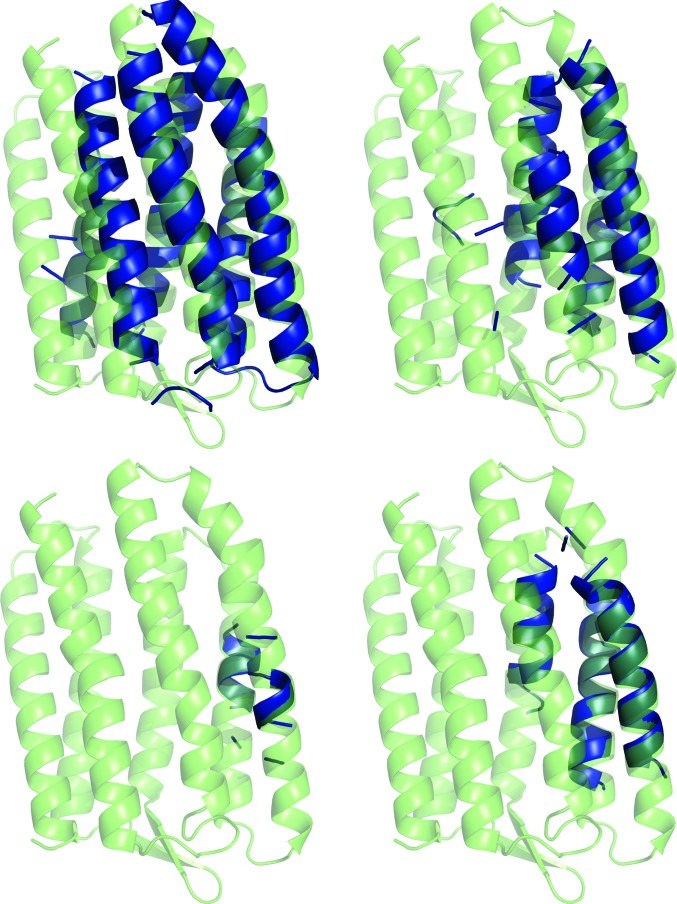
Successful search models (blue) overlaid on the crystal structure of PDB entry 1gu8 (green). Clockwise from top left: c1_t70_r2_polyAla (167 residues), c1_t40_r2_polyAla (95 residues), c1_t25_r1_polyAla (59 residues) and c1_t10_r1_allatom (23 residues).

Solution was then attempted with models generated by the *GREMLIN* modelling protocol. The results are displayed in Fig. 1 ▸, with a graphical summary shown in Supplementary Fig. S6 and an analysis of the TM scores shown in Supplementary Fig. S7. *GREMLIN* was able to solve four targets, including 1gu8, which could not be solved with *AMPLE*’s simple approach to ideal helices, *QUARK* or *ROSETTA­MEMBRANE*. However, targets 3ldc, 3ouf and 2o9g were not solved, all of which could be solved with the ideal helices, and neither was 3gd8, which could be solved with *ROSETTAMEMBRANE*. The contact information used by *GREMLIN* dramatically improved the quality of the models, so that the median TM score across all models was 0.667, as opposed to 0.263 for *ROSETTAMEMBRANE* and 0.385 for *QUARK*. It also appears that the better models are contributing more to the solutions. 20 successful models were generated for target 1gu8, with a wide range of model sizes (23, 35, 47, 59, 71, 83, 95, 107, 119 and 167 residues). A selection of four of the successful solutions, covering the whole span of sizes, is shown in Fig. 4 ▸.

Fig. 4 ▸ shows that all of the solutions were derived from the same cluster, with the smaller ensembles being heavily truncated versions of the larger ones. The models for target 1gu8 are much better than with any of the previous methods (a maximum TM score for successful/unsuccessful of 0.829/0.856) and this is what appears to have made solution possible. The modelling has captured the packing and helical curvature of six of the helices. It is interesting that for the smallest solution (c1_t10_r1_allatom with 23 residues), just two short helical segments correctly packed against each other are sufficient to elicit a solution, whereas an ideal helical segment of the same length cannot. Interestingly the ensemble derived from the untruncated cluster of models, the centroid of which had a TM score of 0.856, was unable to solve the target.

### 3.5. *CCMPRED*, *MEMBRAIN* and *MetaPSICOV_S*1   

Solution was then attempted with models built with the assistance of contact predictions from *CCMPRED*, *MEMBRAIN* or *METAPSICOV_S*1. The results are summarized in Fig. 1 ▸, with graphical summaries of the results shown in Supplementary Figs. S8, S10 and S12, and analyses of the TM scores shown in Supplementary Figs. S9, S11 and S13.

The successes across the three modelling protocols were mixed. *CCMPRED* solved three targets, *MEMBRAIN* solved five and *METAPSICOV_S*1 solved four; thus, none solved as many targets as the ideal helix run. However, *CCMPRED* and *METAPSICOV_S*1 both solved target 4dve, which could not be solved with any other method, and *METAPSICOV_S*1 also solved target 2o9g, which had previously only been solved with the *AMPLE* library of ideal helices.

**Figure 5:**
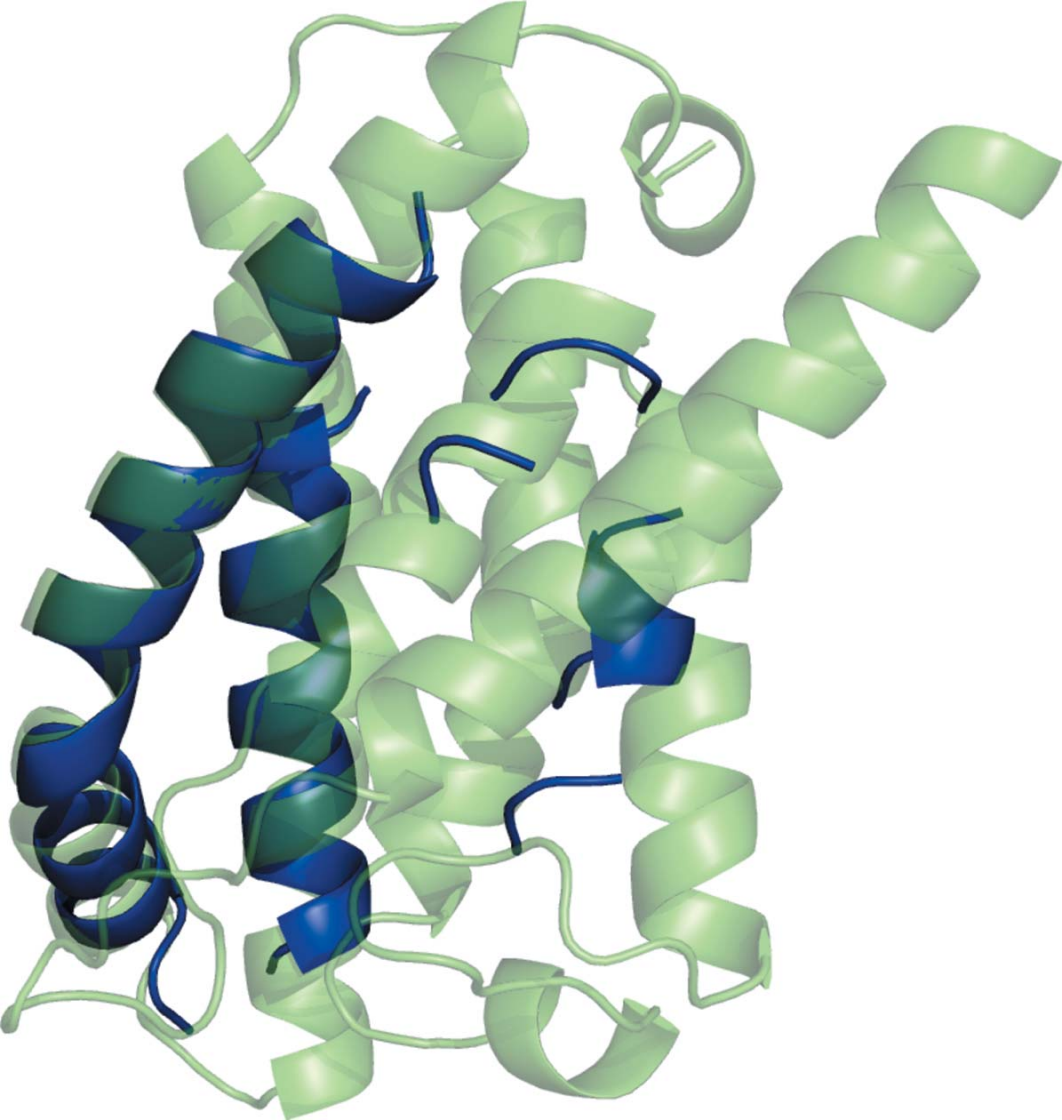
Successful search model c1_t34_r1_allatom from the *CCMPRED* run in blue overlaid on the crystal structure of PDB entry 4dve in green.

Figs. 5 ▸ and 6 ▸ show the successful search models for target 4dve. It can be seen that for the *CCMPRED* solution c1_t34_r1_allatom the modelling has created a kinked helix that has captured the packing and alignment of a slightly curved helix with a shorter helix that follows it. For the *METAPSICOV_S*1 search model c1_t90_r3_polyAla, the modelling performed extremely well (TM score of 0.7201 for the full model used as the ensemble centroid) and has captured both the overall packing and curvature of the helices.

### 3.6. Analysis of model quality   

**Figure 7:**
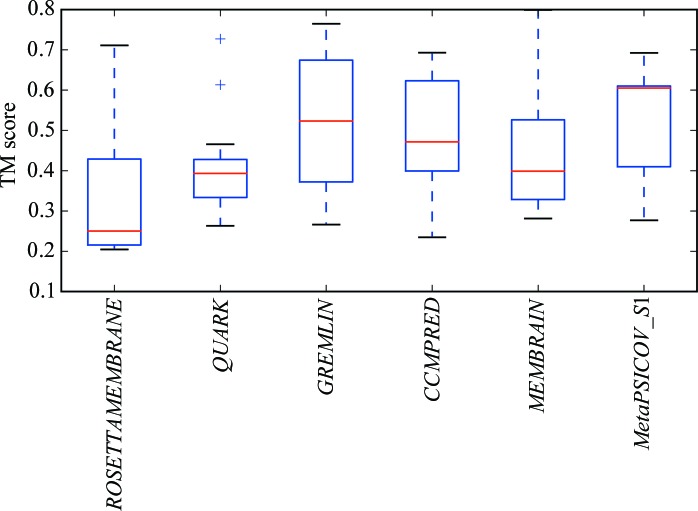
Boxplot of the distribution of TM scores of the models from the top cluster for all targets across each type of modelling run. For each distribution, the red line indicates the median value, the upper and lower edges of the blue rectangle indicate the first and third quartile values, respectively, and the the black horizontal lines represent the minimum and maximum values with the exception of outliers, which are shown as crosses.

A boxplot of the overall TM scores for all the different modelling runs is displayed in Fig. 7 ▸.

The plot shows that there is a very wide variation in the quality of the models produced by all of the modelling protocols. However, it is clear that the contact-assisted models (*GREMLIN*, *CCMPRED*, *MEMBRAIN* and *Meta­PSICOV_S*1) are generally better than those without contact information (*ROSETTAMEMBRANE* and *QUARK*).

### 3.7. Analysis across all runs   

When looking across all targets and all runs, as displayed in Fig. 1 ▸ and Supplementary Figs. S14 and S15, it is clear that resolution plays an important factor, although the modelled chain length and number of residues also influence success to some extent. It is not surprising that the target with the lowest resolution and the largest number of residues in the unit cell (PDB entry 2bhw) could not be solved at all.

**Figure 8:**
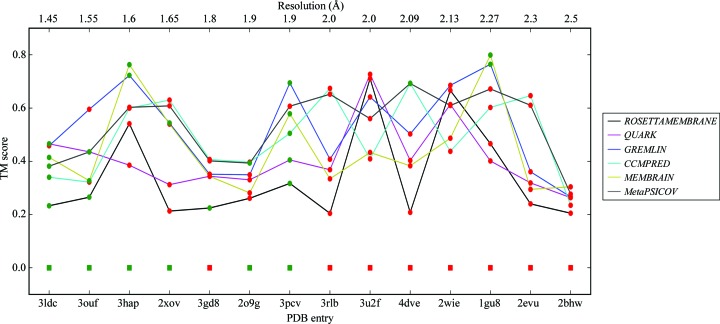
Plot of the median TM scores of the models in the top cluster for the different targets ordered by resolution. Points are coloured green if the target was solved and red otherwise. The ideal helix solutions are plotted as squares along the bottom with a TM score of 0.0.

Analysing the successes and failures against the median TM score for the centroid model of the top cluster from *SPICKER* for the different runs, with the targets ordered by resolution, in Fig. 8 ▸ sheds some light on the performance of the various modelling protocols. It shows that improved modelling as indicated by the TM score is not indicative of a better chance of MR success and that instead resolution is the dominant parameter. Almost all structures with a resolution of better than 2.0 Å were solved with the library of ideal helices. The notable exception is 3gd8, which has been addressed earlier. For structures with a resolution poorer than 2.0 Å, good modelling is occasionally able to generate models from which *AMPLE* can create successful search models. Notable examples are targets 4dve and 1gu8, where solution only becomes possible when the TM score rises to 0.69 and 0.8, respectively. For targets 3u2f, 3rlb and 2wie solution is never achieved, despite the *QUARK* models for 3u2f achieving a median TM score of 0.79, the *CCMPRED* models for 2wie achieving a TM score of 0.744 and the GREMLIN models for 3rlb achieving a TM score of 0.78.

That targets 3u2f, 3rlb and 2wie were not solved despite reasonable models being generated could be an indication that the crystallographic data were particularly poor. However, an analysis of crystallographic quality metrics in Supplementary Table S3, such as the redundancy, *R*_merge_ and *I*/σ(*I*), did not indicate that any of these targets differed systematically from the others. An example of such an analysis is Supplementary Fig. S16, which shows the TM score plotted against the *I*/σ(*I*) of the highest resolution shell and demonstrates that there is no particular pattern between the data quality as measured by this metric, the quality of the models and what could be solved.

**Figure 9:**
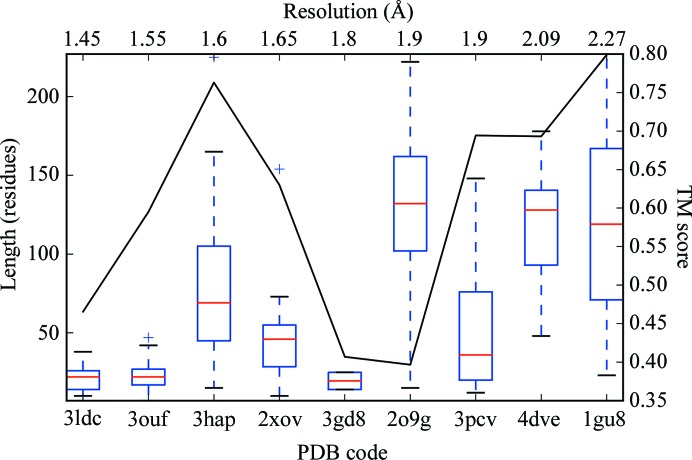
Boxplot of the size of successful search models for the different targets ordered by resolution. The maximum TM score for the ensemble centroid model is also plotted for comparison.

It is interesting to examine any correlation between the quality of the modelling and the size of the search model that is able to solve a structure. It would be expected that better modelling would enable larger search models to be successful, whereas as the modelling became poorer more of the model would be need to be truncated away to leave an accurate core that could facilitate solution. Fig. 9 ▸ overlays a plot of the maximum TM score of the ensemble centroid model for all successful ensembles on a histogram showing the size distribution of successful models.

**Figure 10:**
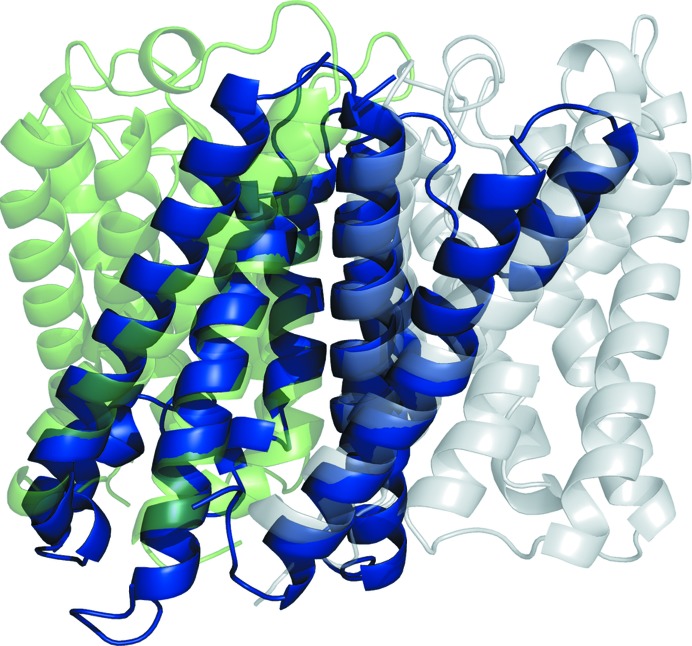
Search model from ensemble c1_t95_r3_reliable in blue overlaid on the crystal structure of PDB entry 2o9g in green, with the symmetry mate of 2o9g in grey: side view.

The plot shows that there is a good correlation between the quality of the models and the size of successful models, as expected. A notable exception is 2o9g, which was solved with a search model of size 234 residues (95% of the length of the full target), despite the full ensemble centroid model having a relatively poor TM score of 0.42, indicating that the overall fold has not been accurately modelled. An examination of this unexpected success revealed an interesting explanation. Analysis of the largest successful search model for target 2o9g, which was from the ensemble c1_t95_r3_reliable generated by *MetaPSICOV* modelling, showed that the search model was placed in the unit cell so as to partially overlap both with the crystal structure and with one of its symmetry mates, as shown in Figs. 10 ▸ and 11 ▸.

Although the search model is a poor match for the crystal structure, the overlap with the symmetry mate places the search model so as to overlap with almost the entire content of the asymmetric unit. Supplementary Fig. S17 shows the search model placed so that the section that overlaps the symmetry mate has been wrapped onto the original copy of the crystal to demonstrate the full extent of the overlap. This shows that the search model is an excellent match for the overall crystal structure, as demonstrated by a TM score of 0.77 for the wrapped model with the crystal structure. Target 2o9g contains a monomer of an aquaporin membrane channel, the biological assembly of which is a homotetramer of four subunits related by a fourfold symmetry axis. As shown in Supplementary Fig. S18, the search model sits perfectly on the interface between two monomers.

Where a protein is active as an obligate oligomer, evolutionary covariance will emerge in an intermolecular fashion at the subunit interfaces. Since there are no reliable methods to distinguish between intramolecular and intermolecular contact pairs in homo-oligomers, and the latter were also present in the sets used to drive modelling, the folding will try to satisfy all corresponding restraints. The aquaporin chain contains two subdomains that are structurally similar and are considered to have arisen by evolutionary duplication (Park & Saier, 1996 ▸). The *METAPSICOV_S*1 model represents two subdomains spanning a subunit interface, rather than two subdomains within a single chain, yet the accurate interface packing unexpectedly captured by the modelling allows successful structure solution.

In order to test our hypothesis that intermolecular contact predictions drove the successful *METAPSICOV_S*1 modelling, we compared the set of predictions against the crystal structure. A comparison of the predicted *METAPSICOV_S*1 contact pairs against the contact pairs extracted from the monomer at 8 Å distance between C^β^ and C^β^ (C^α^ in case of Gly) atoms indicated a high precision of 62.4%. Indeed, looking only at the dimer interface, 21 contacts in the top *L* pairs were predicted correctly with an average confidence score of 0.435. In particular, two hotspot residues alone, 45 and 102, cover seven correctly predicted contacts (Supplementary Fig. S19). Similarly, in the *CCMPRED* top *L* contact pairs, 23 contacts were found in the dimer interface, although the corresponding models were unsuccessful. The *MEMBRAIN* server did not predict a single contact pair across the dimer interface.

The placing of a search model so as to overlap with a symmetry mate is something that has been observed previously with *AMPLE*. In part, it was inaccurate positioning of long helical fragments so as to overlap with a neighbouring symmetry mate in the first *AMPLE* paper (Bibby *et al.*, 2012 ▸) that prompted our work on coiled-coil proteins (Thomas *et al.*, 2015 ▸), where we also observed this phenomenon. In these cases, though, it was largely the fortuitous placement of a fragment that happened to cross the boundary of the asymmetric unit that facilitated solution, although correct generic helical packing modes were correctly captured. The solution of target 2o9g is different as the addition of the contact information has explicitly resulted in the modelling, albeit in an intramolecular fashion, of the intermolecular interface.

## 4. Discussion   

This exploration of the ability of *AMPLE* to solve α-helical transmembrane proteins was prompted by our earlier successes solving small globular proteins, where 80% of the entirely α-helical structures could be solved (Bibby *et al.*, 2012 ▸), and with coiled-coil proteins (Thomas *et al.*, 2015 ▸), where again 80% of the structures could be solved. A notably positive outcome of this work is the solution of all bar one of the targets with a resolution of better than 2.0 Å using a small library of eight ideal helices.

Using ideal helical fragments to solve structures has been demonstrated before by programs such as *ARCIMBOLDO_LITE* (Sammito *et al.*, 2015 ▸), and a transmembrane structure was solved with helical fragments using *ARCIMBOLDO_BORGES* (Sammito *et al.*, 2013 ▸; Millán *et al.*, 2015 ▸), for example. However, these approaches use a more sophisticated MR procedures than *AMPLE* does, and *ARCIMBOLDO_BORGES* employs a curated library of fragments derived from existing structures. That a simple MR approach with a small library of ideal helices can solve so many of this test set is encouraging, as it shows that neither laborious experimental phasing nor relatively computationally expensive modelling or MR protocols may be required to solve transmembrane proteins with a resolution better than 2.0 Å.

For the current target set, where the resolution is poorer than 2.0 Å some form of *ab initio* modelling is required in order to generate a sufficiently large search model to enable solution. The two contact-free modelling protocols, *ROSETTA­MEMBRANE* and *QUARK*, were able to generate solutions, but the overall quality of the modelling was poor and the protocols could only solve one structure that could not be solved with *AMPLE*’s set of ideal helices.

The addition of inter-residue contact pairs results in a marked increase in the overall quality of the models and facilitates the solution of two structures that could not be solved using any other protocol. These two structures were both at moderate resolution (2.09 Å for target 4dve and 2.27 Å for target 1gu8), and target 4dve, with 594 residues, was the second largest by number of residues in the asymmetric unit. This improvement of the modelling when contact-prediction information is included is especially encouraging, as contact prediction is a fast-moving field that is evolving and improving rapidly: deep learning, for example, seems to hold particular promise (Wang *et al.*, 2017 ▸), and metagenomic data will inevitably spread the availability of this information across more protein families (Ovchinnikov *et al.*, 2017 ▸). Transmembrane proteins seem to offer both particular challenges and opportunities. On the one hand their low packing densities and water-containing cavities are likely to weaken the covariance signal that contact prediction relies on (Rose *et al.*, 2014 ▸) and the relatively limited number of transmembrane-protein structures available limits the accuracy with which biophysical parameters relevant to methods development can be determined (Li *et al.*, 2017 ▸). On the other hand, the fundamentally limited range of intramembrane packing interactions facilitates the development of bespoke transmembrane-protein methods of contact prediction (see, for example, Li *et al.*, 2017 ▸). For the same reason, there are good reasons to think that specific transmembrane-protein *ab initio* modelling protocols will produce better results than general methods. It is unfortunate that the one attempted here (*ROSETTAMEMBRANE*) does not appear to be being actively developed, but other methods are in development (Law *et al.*, 2017 ▸).

A particularly interesting feature of the contact modelling was the generation of a homodimer interface for the target 2o9g by *METAPSICOV_S*1. Although this facilitated the solution of target 2o9g, it demonstrates the current inability of contact-generation algorithms to separate the intramolecular and intermolecular contacts. Although either set of contacts can be useful for different purposes (Simkovic, Ovchinnikov *et al.*, 2017 ▸), until it becomes possible to separate them the modelling will attempt to satisfy both sets at the same time, which can confidently be predicted to result in poorer models than if a single set were used. Curiously, high-quality models with TM scores of around 0.8 were calculated for this target, but clusters containing them were not sources of successful search models.

As well as highlighting the value of a putative method to distinguish intramolecular and intermolecular contact predictions, the results offer other pointers towards future productive algorithmic developments. When the modelling performs well, *AMPLE* is often able to generate an ensemble that can solve the structure; this applies to both high-resolution and low-resolution structures. With high-resolution structures, *SHELXE* is able to trace up to a full structure, even when only a small fragment has been placed correctly, as is the case for ideal helices. With high-resolution structures, when the modelling is relatively poor, *AMPLE* must prune away enough of the incorrect structure to leave a sufficiently accurate substructure suitable for MR and tracing with *SHELXE*. Sometimes the truncation algorithm in *AMPLE* is able to perform this (examples include targets 3ouf and 2xov), although at other times it fails and the truncated ensemble is unable to solve the structure, whereas an ideal helix of the same length can. In these cases, it will be of value to explore whether bespoke truncation protocols for transmembrane proteins could improve performance.

All of this work was undertaken with *CCP*4 v.6.5.3 (including *SHELXE* v.2014/14 and *ARP*/*wARP* v.7.5), which was released on 3 July 2015. This was necessary to ensure that the different runs of *AMPLE* with the different modelling protocols were comparable. As of the time of writing *CCP*4 is at v.7.0.045 (including *SHELXE* v.2017/1) and considerable improvements have been made in the *CCP*4 software packages that *AMPLE* relies on. In related work (unpublished) we have run a number of the cases in this work with a newer version of *CCP*4 and observed significant improvements in our success rate. We therefore expect that a user of *AMPLE* using the very latest version of *CCP*4 would have an even better chance of solving their structure than the work in this paper suggests.

In summary, this work shows that *AMPLE* can already solve many (9/14) of a set of α-helical transmembrane proteins. Higher resolution cases can be attempted first using ideal helices, while others yield to the ability of *AMPLE* to construct MR search ensembles from *ab initio* models. Targets for which contact predictions can be calculated show a distinct benefit from their use. As data volume and methods development push contact prediction to better performance in the future, the overall success rate of *AMPLE* can be expected to improve further.

## Supplementary Material

Supplementary Methods, Tables and Figures.. DOI: 10.1107/S2059798317016436/qh5049sup1.pdf

## Figures and Tables

**Figure 2:**
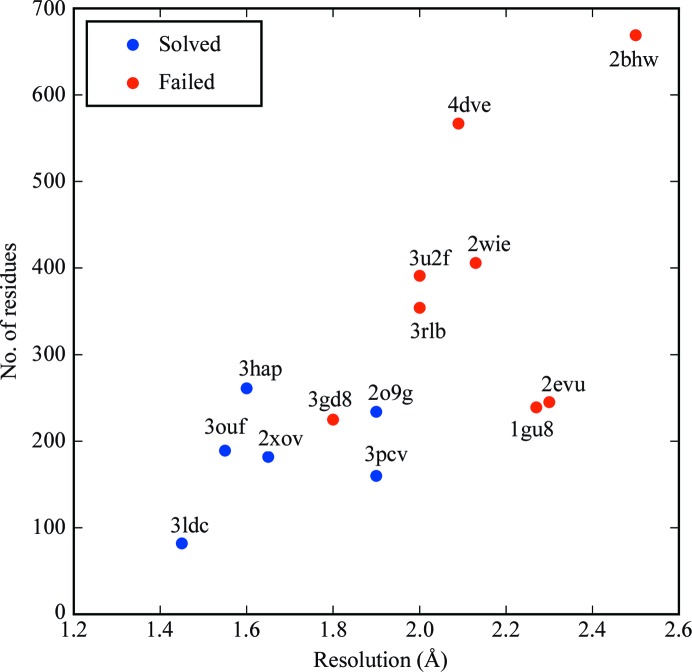
Results for attempting solution of transmembrane proteins with ideal helices mapped against target resolution and number of residues in the asymmetric unit of the crystallographic cell. Successes are in blue and failures are in red.

**Figure 6:**
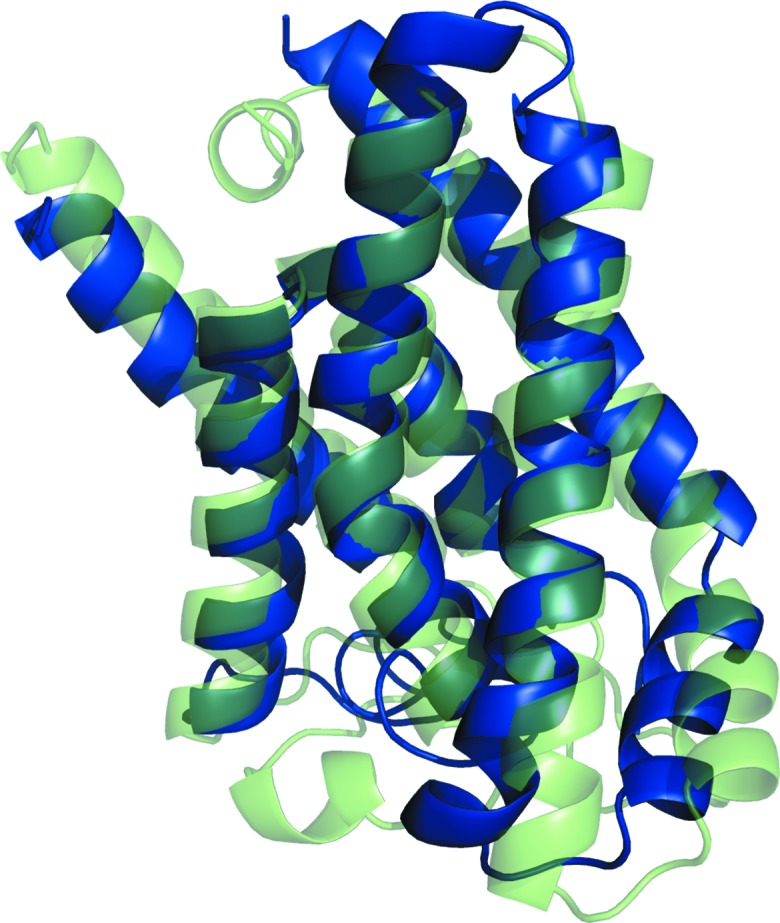
Successful search model c1_t90_r3_polyAla from the *MetaPSICOV_S*1 run in blue overlaid on the crystal structure of PDB entry 4dve in green.

**Figure 11:**
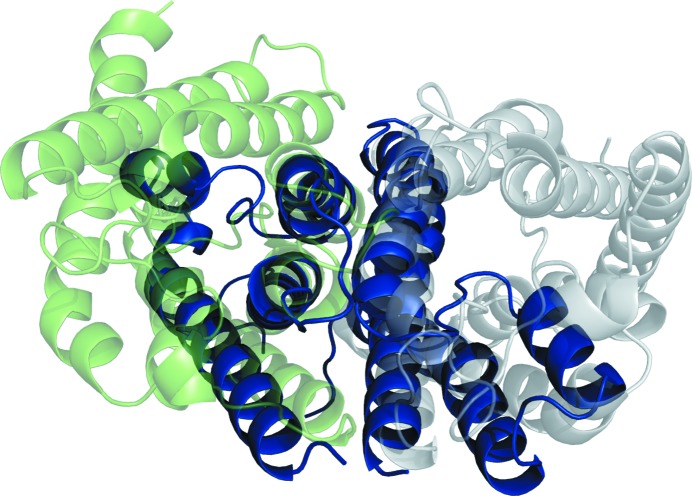
Search model from ensemble c1_t95_r3_reliable in blue overlaid on the crystal structure of PDB entry 2o9g in green, with the symmetry mate of 2o9g in grey: top view.
