# Supplementary material for: Approaches to ab initio molecular replacement of α-helical transmembrane proteins
Source: Acta Crystallogr D Struct Biol. 2017 Nov 22;73(Pt 12):985–96. doi: 10.1107/S2059798317016436 (PMC5713875; doi:10.1107/S2059798317016436)
Supplement: Supplementary file 1 [file d-73-00985-sup1.pdf]

STRUCTURAL BIOLOGY

Volume 73 (2017)

Supporting information for article:

Approaches to *ab initio* molecular replacement of α-helical transmembrane proteins

Jens M. H. Thomas, Felix Simkovic, Ronan Keegan, Olga Mayans, Chengxin Zhang, Yang Zhang and Daniel J. Rigden

S1. Supplementary Methods

S1.1. ROSETTA Modelling with GREMLIN evolutionary contacts

The GREMLIN server (http://gremlin.bakerlab.org/) predicts residue-residue contacts starting from a protein sequence in FASTA format. It generates a multiple sequence alignment with HHblits (Alva *et al.*, 2016*b*) and uses Direct Coupling Analysis methods, like CCMpred (Seemayer *et al.*, 2014*b*), to predict contact pairs. Besides the raw contact prediction output, the GREMLIN server also provides ROSETTA-formatted Cβ-Cβ distance restraints. All contact pairs with a scaled score (raw score / average(raw scores)) of greater than 0.0 are included and formatted to use the SIGMOID energy function in ROSETTA.

The only change required to the standard AbInitioRelax protocol is a modification of the ROSETTA energy function to reflect the exposure of non-polar residues in the membrane-spanning regions. This requires the Lazaridis-Karplus solvation energy term weight to be set to zero, and to compensate for the short-range repulsion implicit in the solvation model, the Lennard-Jones repulsive and attractive terms being given equal weights. This requires the creation of a ROSETTA weights file with the following flags:

fa_atr = 0.8
fa_rep = 0.8
fa_sol = 0.0

The weights file is then supplied to ROSETTA with the '**-score:patch**' command-line flag.

The second change is for the normalized GREMLIN score to be multiplied by three to give the contact restraints roughly the same total dynamic range as the ROSETTA energy. This requires the addition of the '**-constraints:cst_weight 3**' and '**-constraints:cst_fa_weight 3**' flags.

Table S1 Successful search ensembles for target 1GU8 generated by the RosettaMembrane run.

Ensemble name	Number of residues	Models in ensemble	centroid TM	RIO score (inregister)
**c1_tl11_r2_allatom**	25	19	0.210	0
**c1_tl11_r3_polyAla**	25	30	0.210	0
**c1_tl6_r2_allatom**	14	30	0.224	0
**c1_tl6_r3_reliable**	14	30	0.227	0

Table S2 **:**HELANAL analysis for the solutions of 3GD8 with RosettaMembrane

The c1_tl6 solutions were placed aligning with residues LEU 191 -> HIS 201, and the two c1_tl11 solutions aligning with SER 188 -> ALA 204. The analysis is of one of the longer solutions (c1_tl11_r3_polyAla) with HELANAL.

PDB	Helix start-end	Len	Twist	n	h	Aver tor	Aover BA#	Max BA#	Radius	rms S	rms L	Geom
1GU8	189-208	20	98.5	3.65	1.5 4	51.0	19.5	64.8	65	.133	.249	K
c1_tl11_r3_polyAla	41-55	15	97.4	3.70	1.4 8	47.8	6.3	11.3	67	.042	.061	C

Twist : Average unit twist of the helix (Deg.).
n : Average number of residues per turn of the helix.
h : Average unit height of the helix (Angstroms).
Aver vtor : Average virtual torsion angle defined by four CA atoms (Deg.).
Aver BA : Average Bending angle between successive local helix axes (Deg.).
Max BA : Maximum Bending angle between succesive local helix axes (Deg.). Residue number and name given in parenthesis
Radius : Radius of curvature in Angstroms (Radius of least square sphere fitted to the local helix origins).
rmsdS : Root Mean Square Deviation for least square sphere fitted to the local helix origins (Angstroms).
rmsdL : Root Mean Square Deviation for least square 3D line fitted to the local helix origins (Angstroms).
Geometry : Overall geometry of the helix, Linear (L), Curved (C), Kinked (K), or unassigned (-).

Table S3 Select crystallographic data quality metrics for the targets.

All crystallographic data was collected from the PDB (Berman, 2000). Any data not present in the PDB was extracted from Table 1 of the relevant publication. The number of effective sequences (Neff), or depth of the multiple sequence alignment is a metric to describe the number of sequences in the alignment diverse enough to effectively contribute to the contact prediction by Direct Coupling Analysis. The algorithm used to compute Neff is implemented in ConKit (Simkovic, Thomas *et al.*, 2017*b*). The final column 'solved' indicates if the target was soluble with any of the methods attempted in this work.

PDB	Resol[n]	Space Group	Neff	Complete-ness	RMergeI	Redundancy	Reflections for Refinement	rFree	rWork	I/SigI	Solved
**3LDC**	1.45	P 4 2_1 2	942	99.3	0.044	6.3	14863	0.202	0.187	1.3	Y
**3OUF**	1.55	I 4	1065	97.2	N/A	6.7	28666	0.215	0.199	1	Y
**3HAP**	1.6	C 2 2 2_1	183	93.9	0.05	6.3	36725	0.192	0.167	2.8	Y
**2XOV**	1.65	H 3 2	1142	99.8	0.06	4.5	36038	0.218	0.192	2.4	Y
**3GD8**	1.8	P 4 2_1 2	851	99.9	N/A	12	23583	0.165	0.16	3.3	Y
**2O9G**	1.9	I 4	836	85.1	0.065	3	23096	0.195	0.166	1.4	Y
**3PCV**	1.9	F 2 3	444	99.9	0.302	9.9	29381	0.198	0.178	2.4	Y
**3RLB**	2	C 1 2 1	374	98.4	0.058	3.7	41123	0.23	0.206	2.5	N
**3U2F**	2	P 4_2 2 2	254	98.1	0.08	8.6	24159	0.216	0.192	3.2	N
**4DVE**	2.09	C 1 2 1	682	98.4	0.059	10.8	49743	0.203	0.185	2.51	Y
**2WIE**	2.13	P 6_3 2 2	288	99.2	0.15	4.7	37792	0.235	0.197	1.7	N
**1GU8**	2.27	C 2 2 2_1	184	99.4	0.139	5.8	13075	0.256	0.23	1.9	Y
**2EVU**	2.3	I 4	820	94.4	N/A	2.1	15208	0.226	0.188	2	N
**2BHW**	2.5	C 1 2 1	229	85.6	0.08	2.8	47867	0.241	0.22	2.7	N

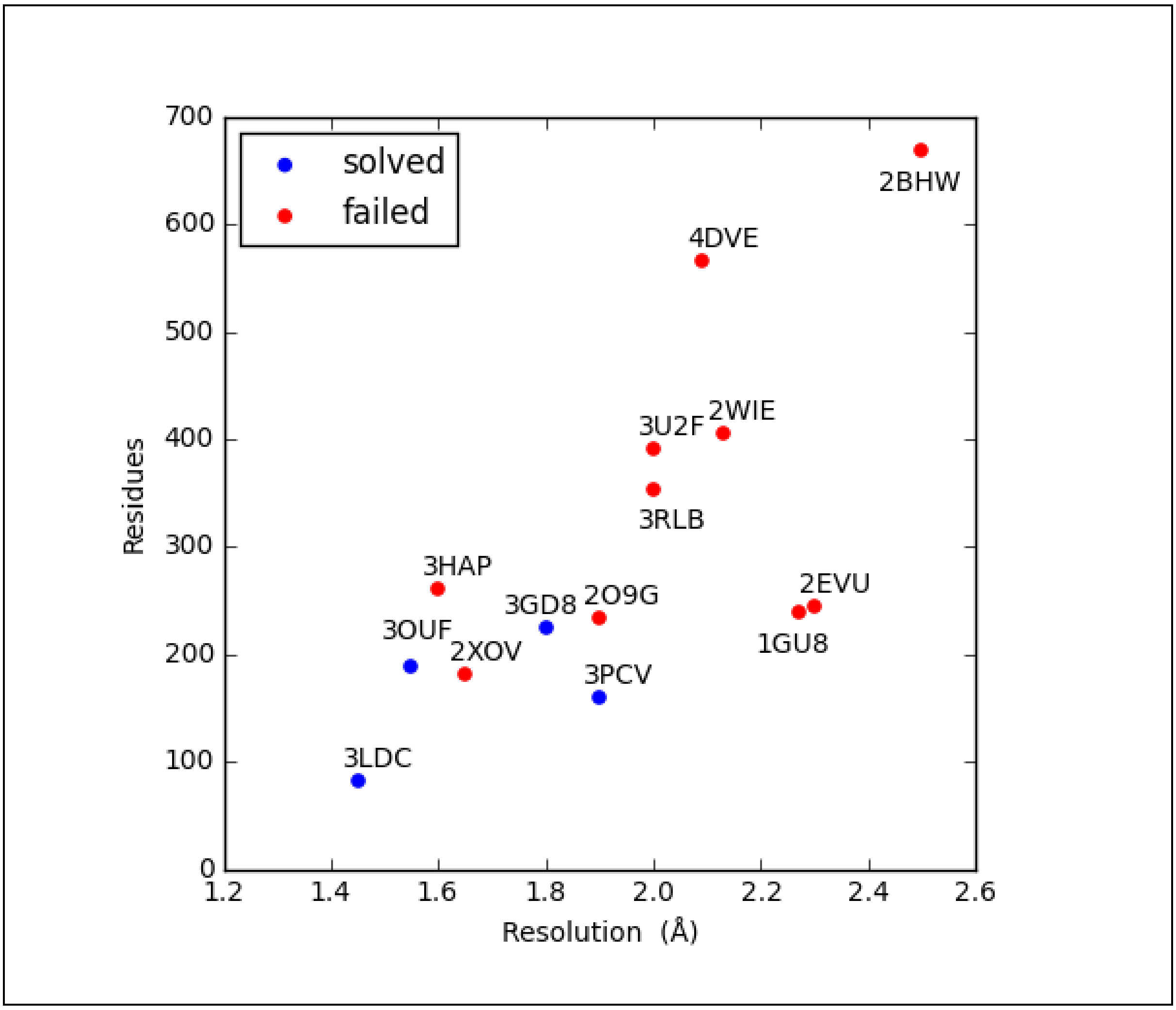

Figure S1 Results for attempting solution of TM proteins with RosettaMembrane, mapped against target resolution and number of residues in the unit cell. Success are in blue, failures in red.

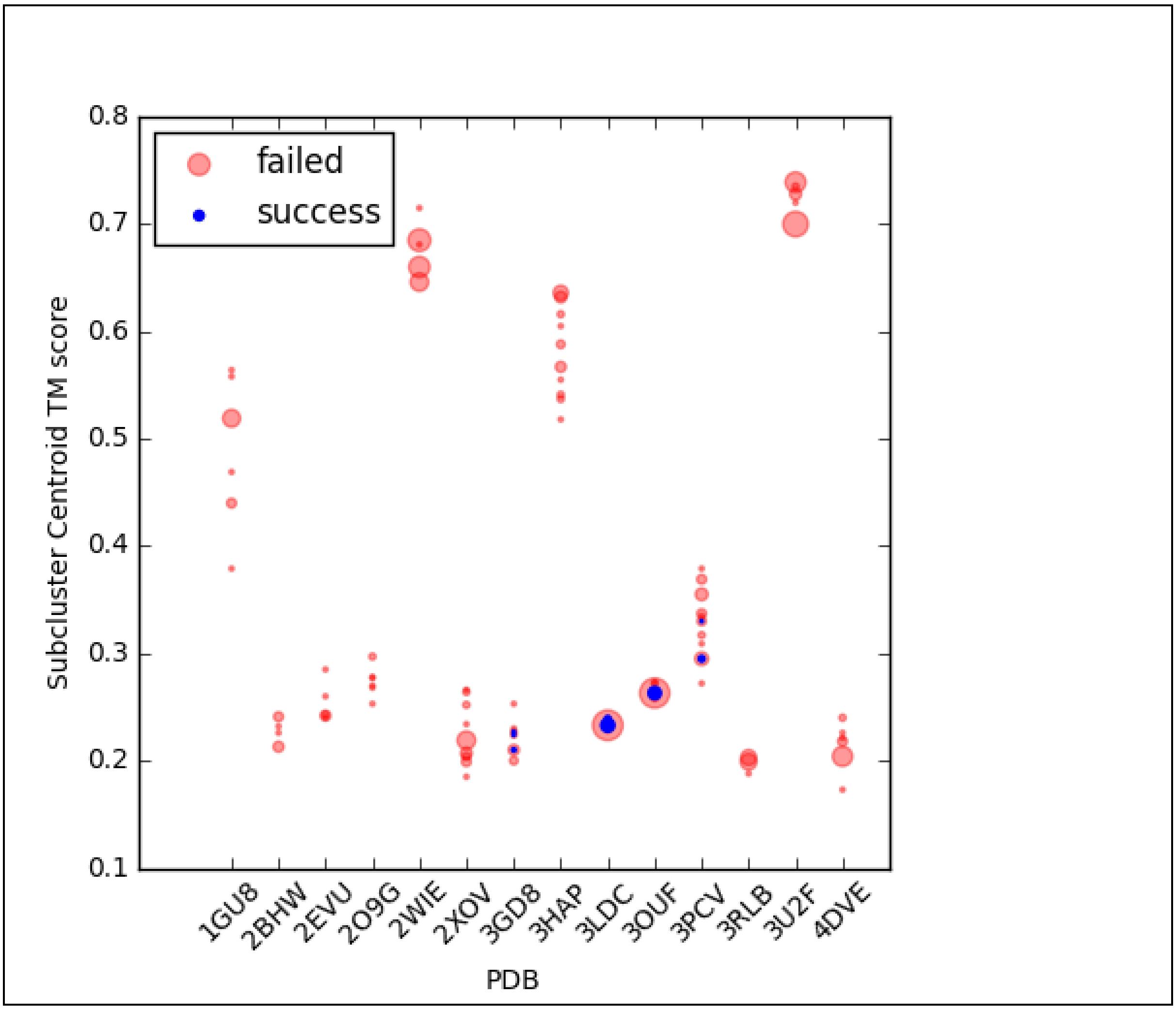

Figure S2 Scatterplot of TM score of the complete (i.e. untruncated) centroid model of the subcluster that was used to form the ensemble, against the target for the RosettaMembrane run. Points are sized by the number of ensembles and coloured red for failing ensembles and blue for successful ones.

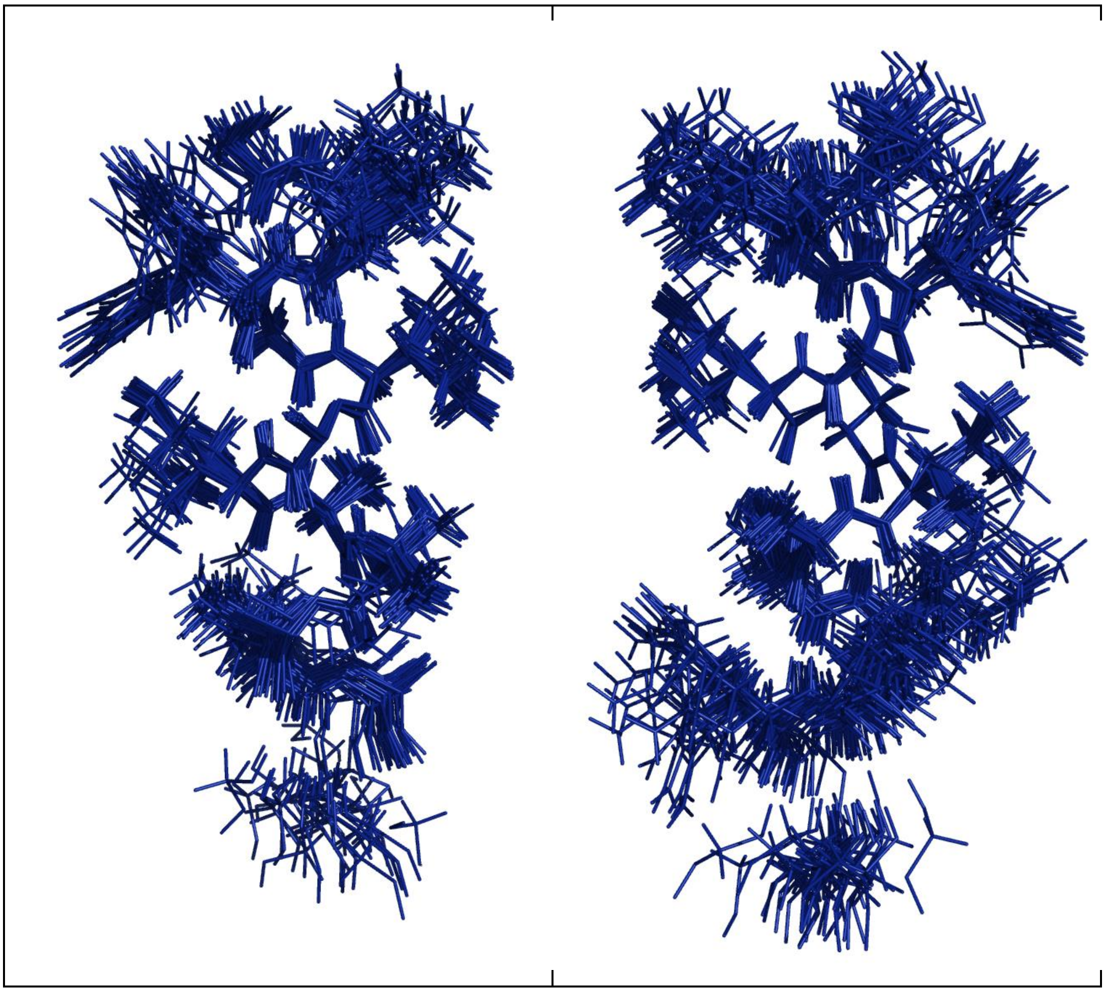

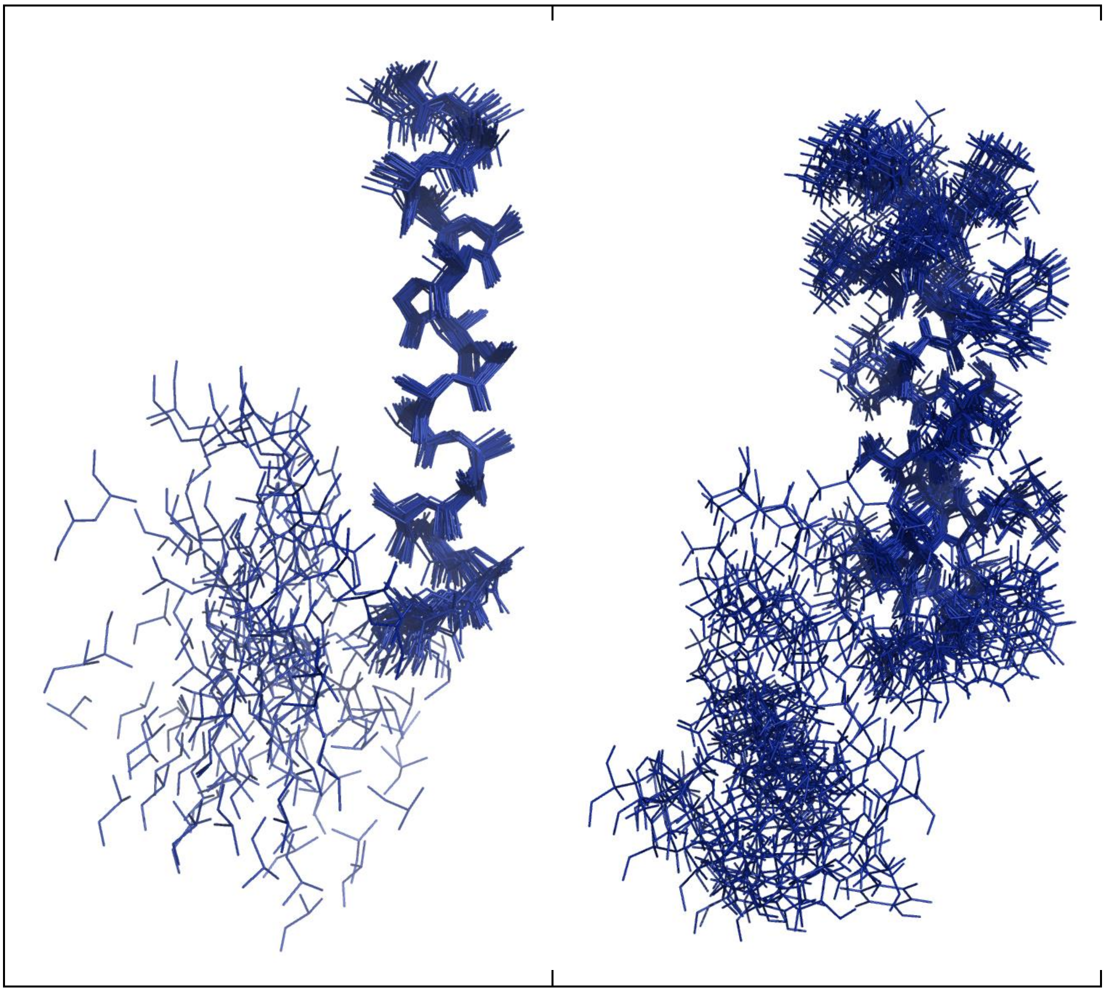

Figure S3 The four ensembles that solved the structure, clockwise from top left: c1_tl6_r2_reliable, c1_tl6_r2_allatom, c1_tl11_r2_allatom and c1_r11_r3_polyAla.

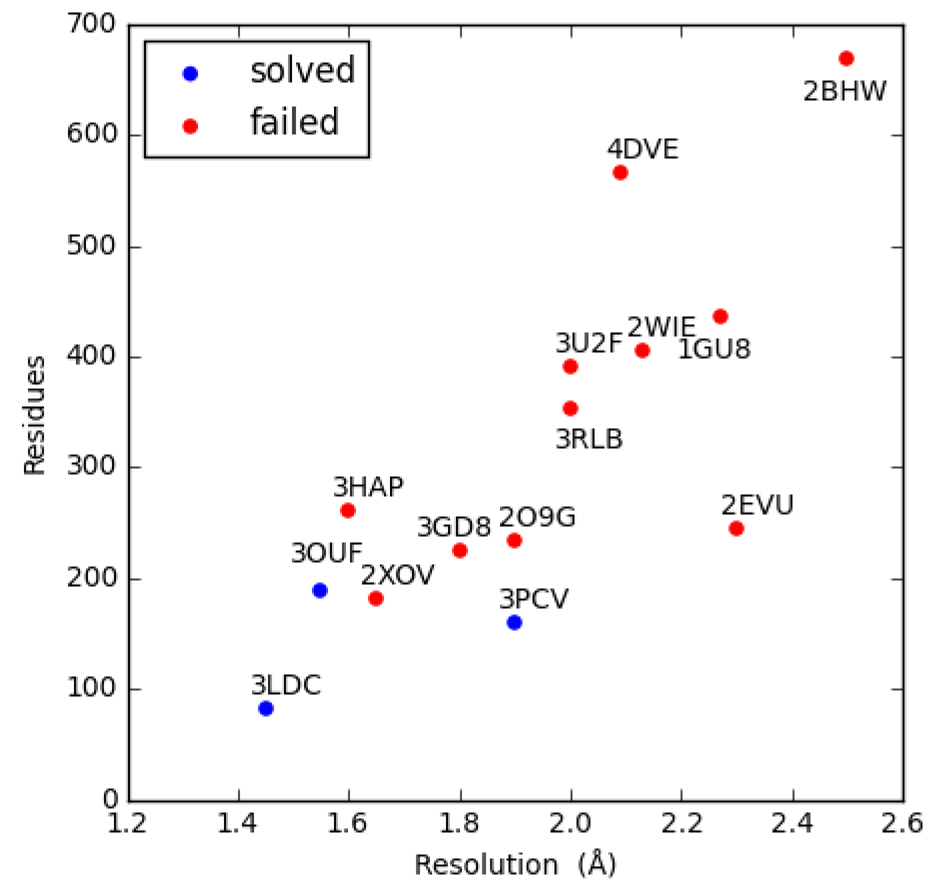

Figure S4 Results for attempting solution of TM proteins with QUARK, mapped against target resolution and number of residues in the unit cell. Success are in blue, failures in red

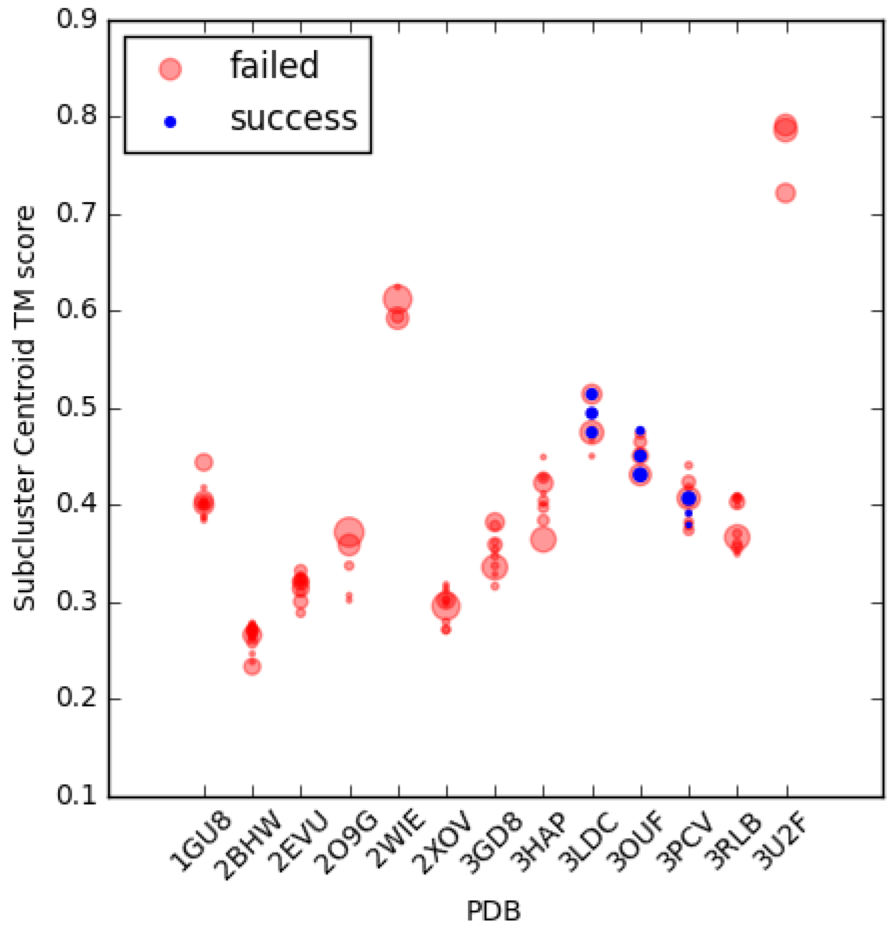

Figure S5 Scatterplot of TM score of the complete centroid model of the subcluster that was used to form the ensemble, against the target for the QUARK models. Points are sized by the number of ensembles and coloured red for failing ensembles and blue for successful ones.

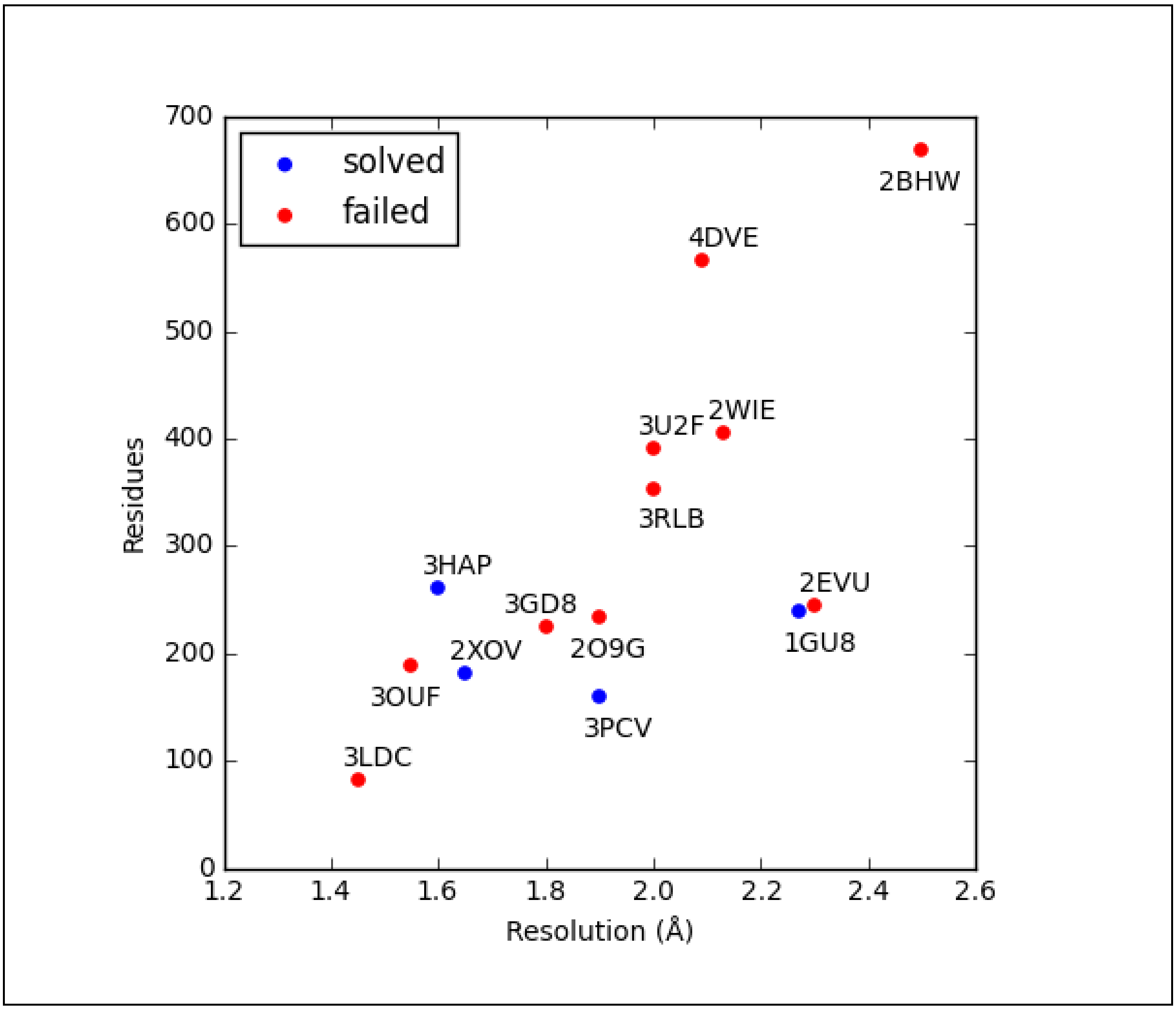

Figure S6 Results for attempting solution of TM proteins with GREMLIN, mapped against target resolution and number of residues in the unit cell. Success are in blue, failures in red.

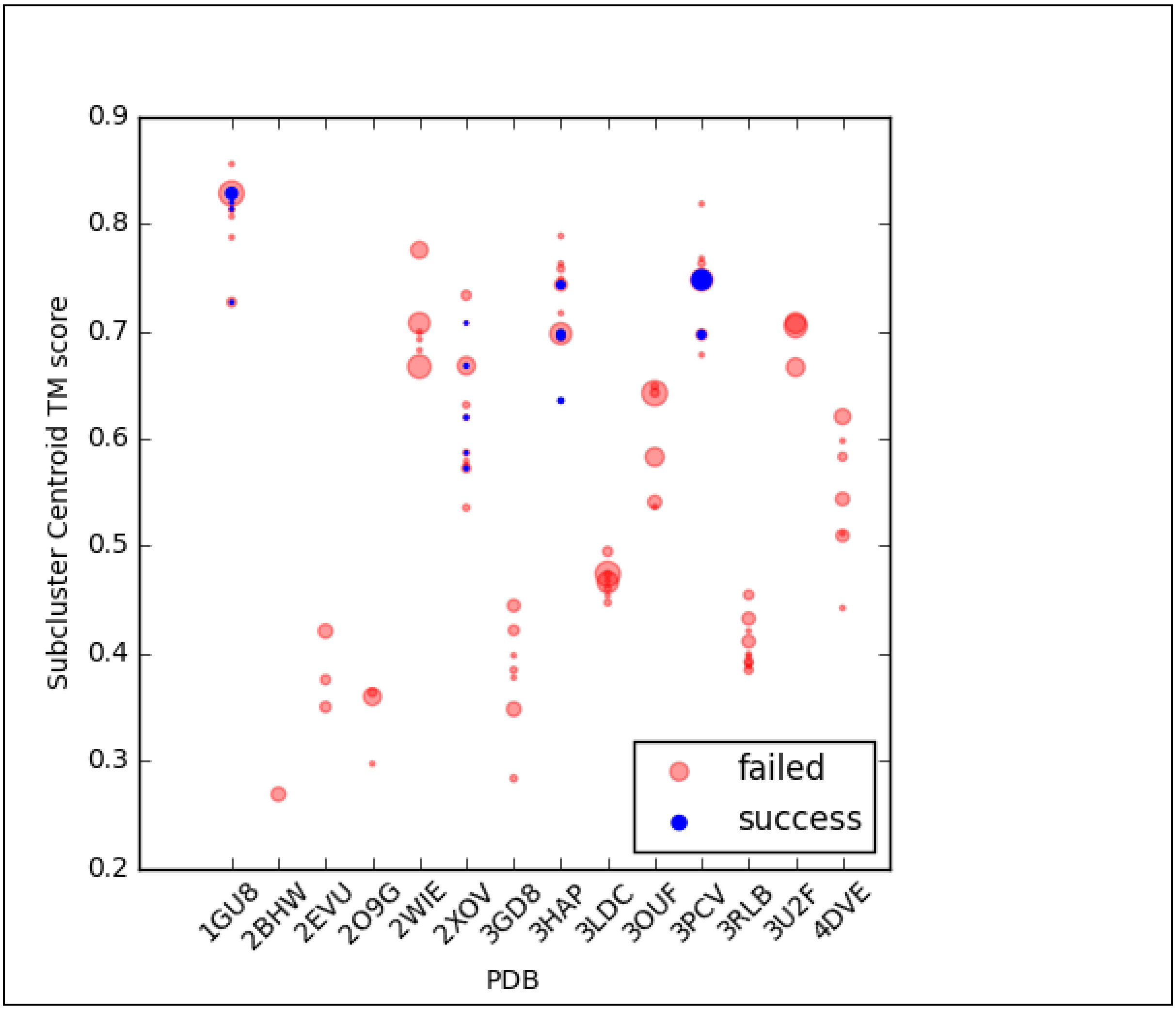

Figure S7 Scatterplot of TM score of the complete centroid model of the subcluster that was used to form the ensemble, against the target for GREMLIN models. Points are sized by the number of ensembles and coloured red for failing ensembles and blue for successful ones.

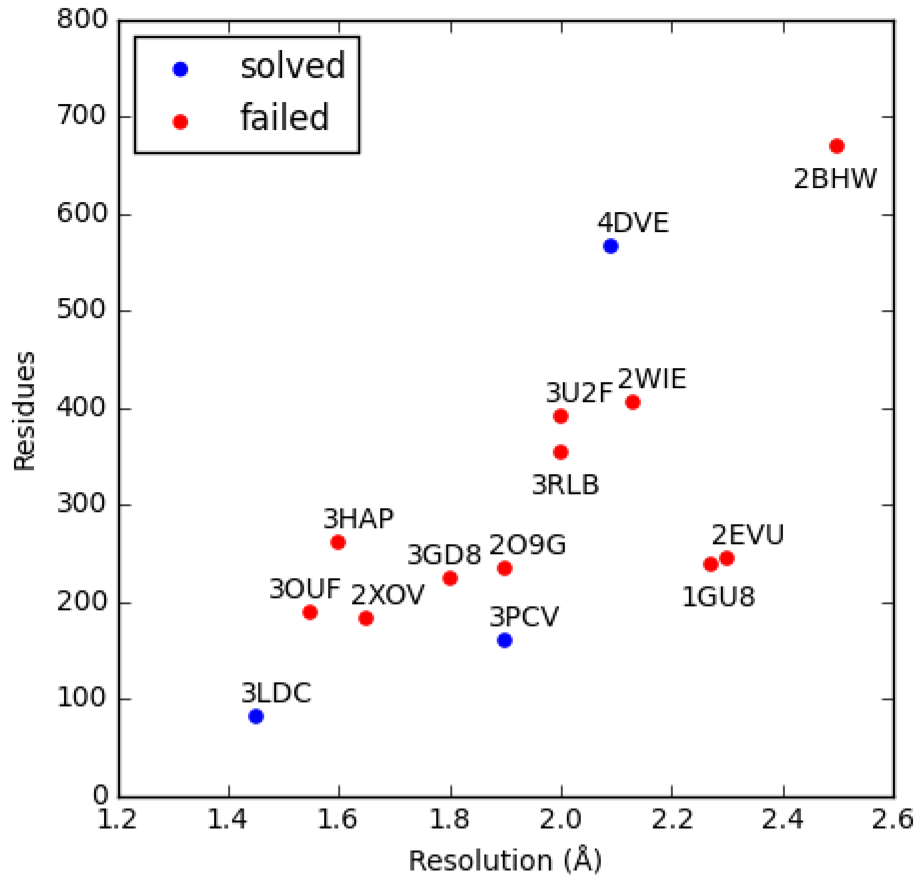

Figure S8 Results for attempting solution of TM proteins with CCMPRED, mapped against target resolution and number of residues in the unit cell. Success are in blue, failures in red.

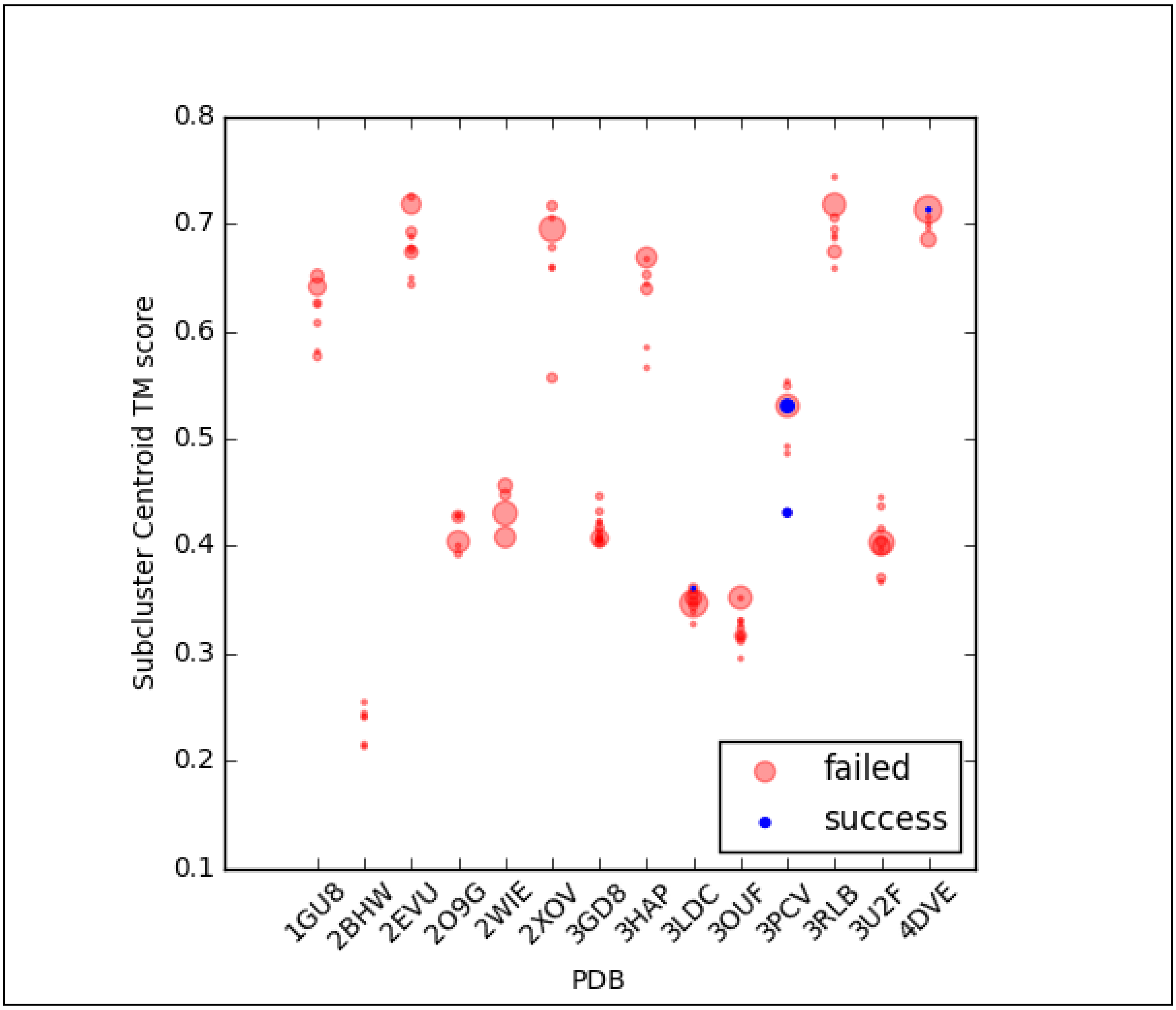

Figure S9 Scatterplot of TM score of the complete centroid model of the subcluster that was used to form the ensemble, against the target for CCMPRED models. Points are sized by the number of ensembles and coloured red for failing ensembles and blue for successful ones.

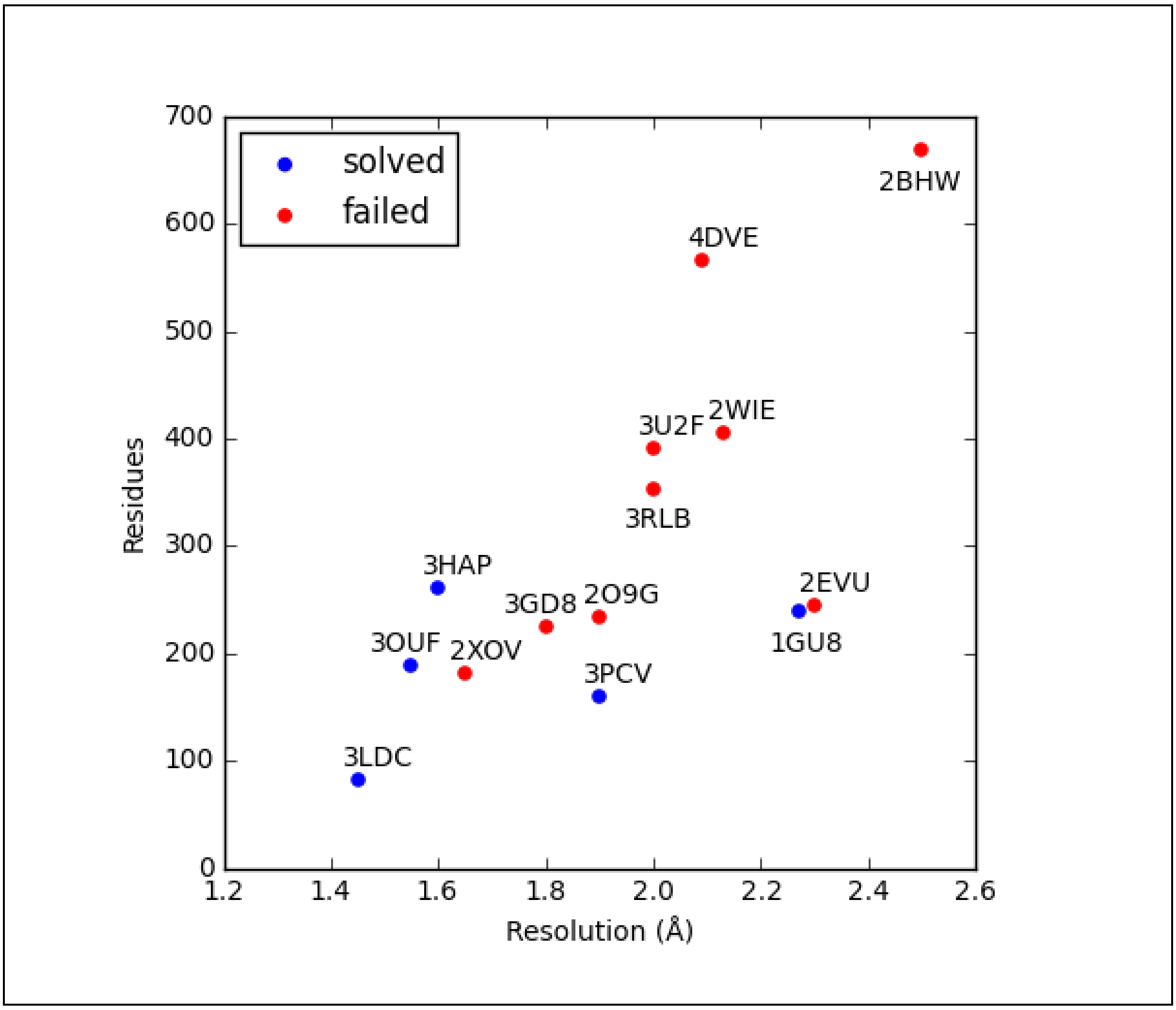

Figure S10 Results for attempting solution of TM proteins with MEMBRAIN, mapped against target resolution and number of residues in the unit cell. Success are in blue, failures in red.

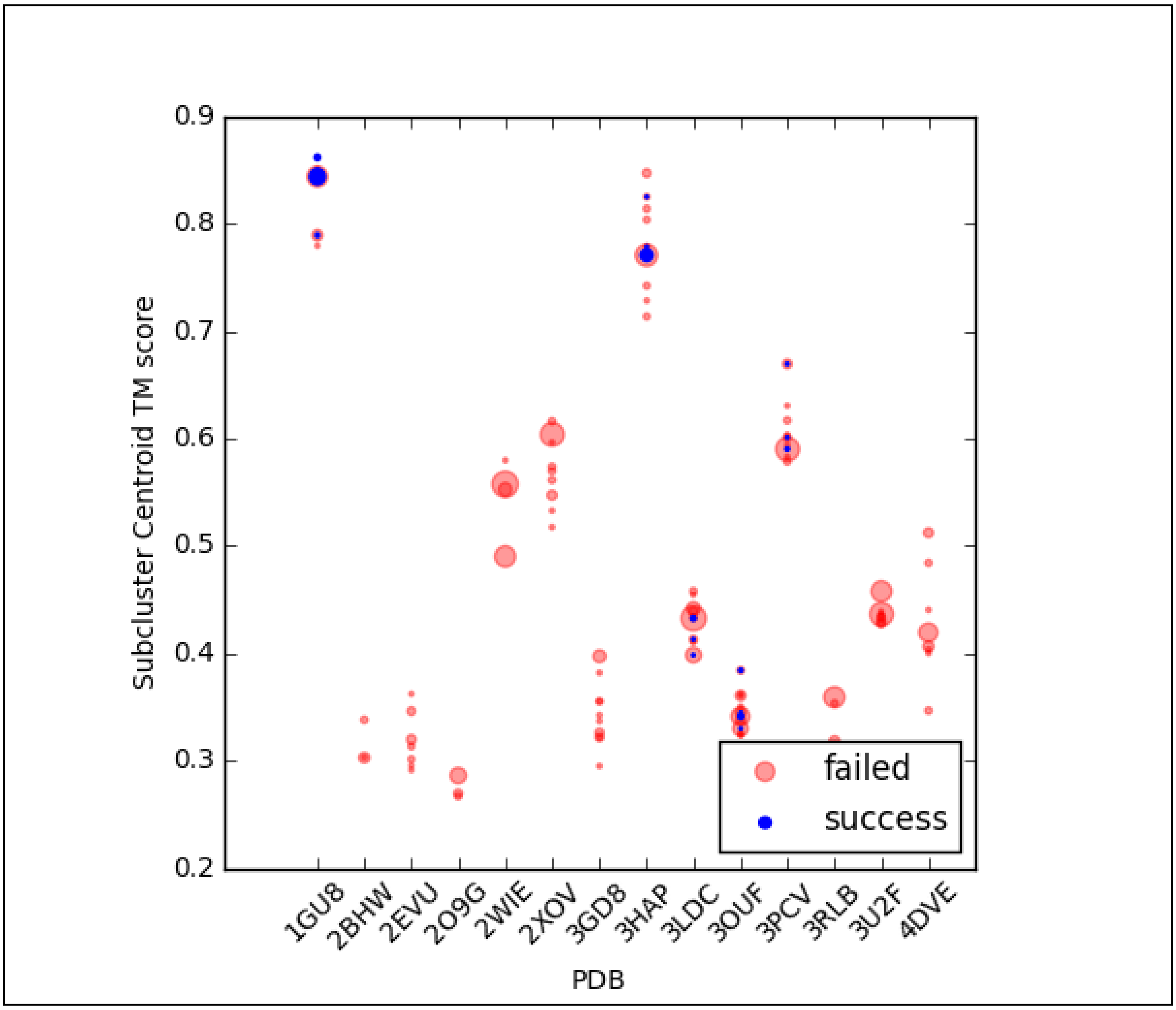

Figure S11 Scatterplot of TM score of the complete centroid model of the subcluster that was used to form the ensemble, against the target for MEMBRAIN models. Points are sized by the number of ensembles and coloured red for failing ensembles and blue for successful ones.

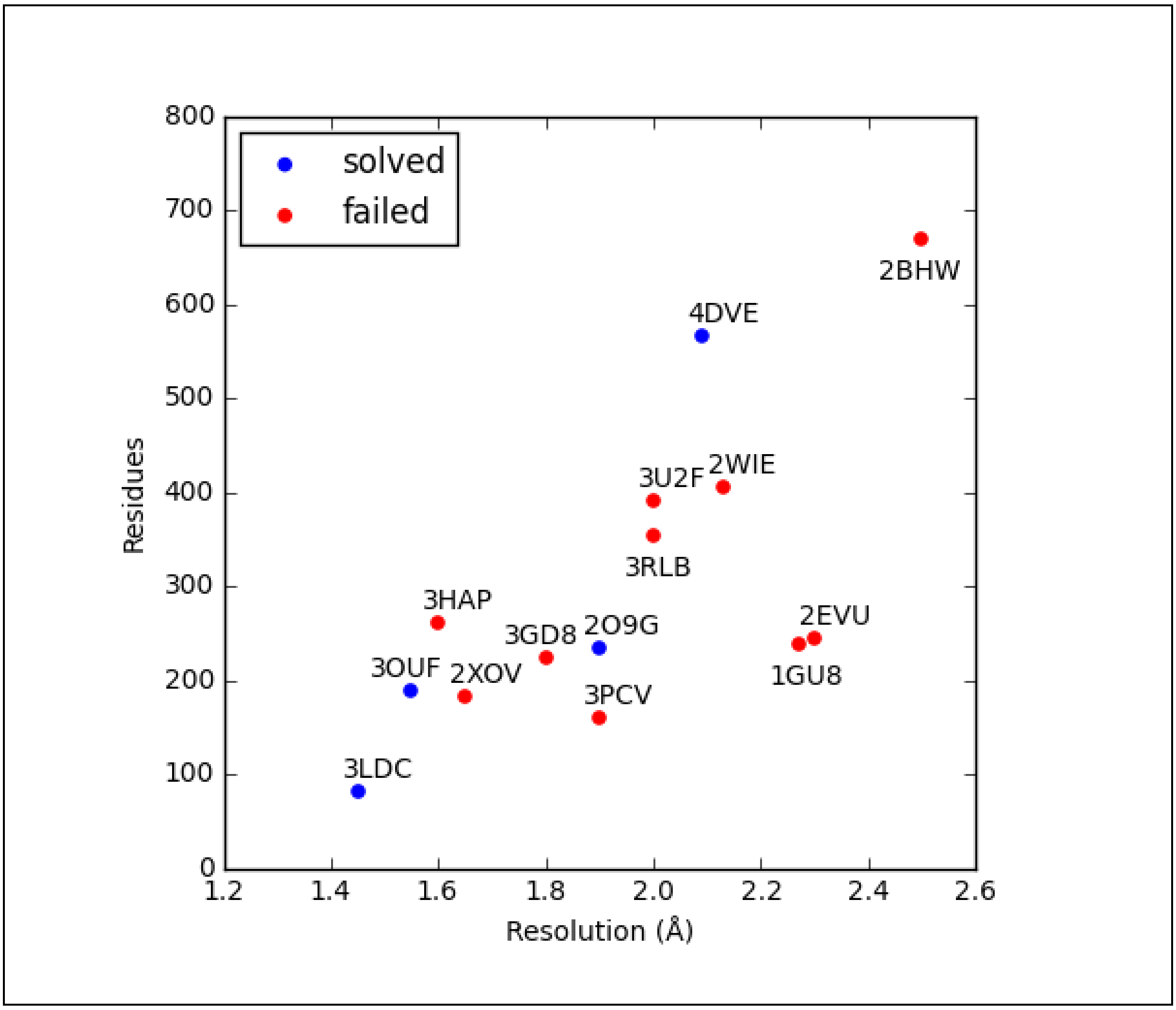

Figure S12 Results for attempting solution of TM proteins with METAPSICOV_S1, mapped against target resolution and number of residues in the unit cell. Success are in blue, failures in red.

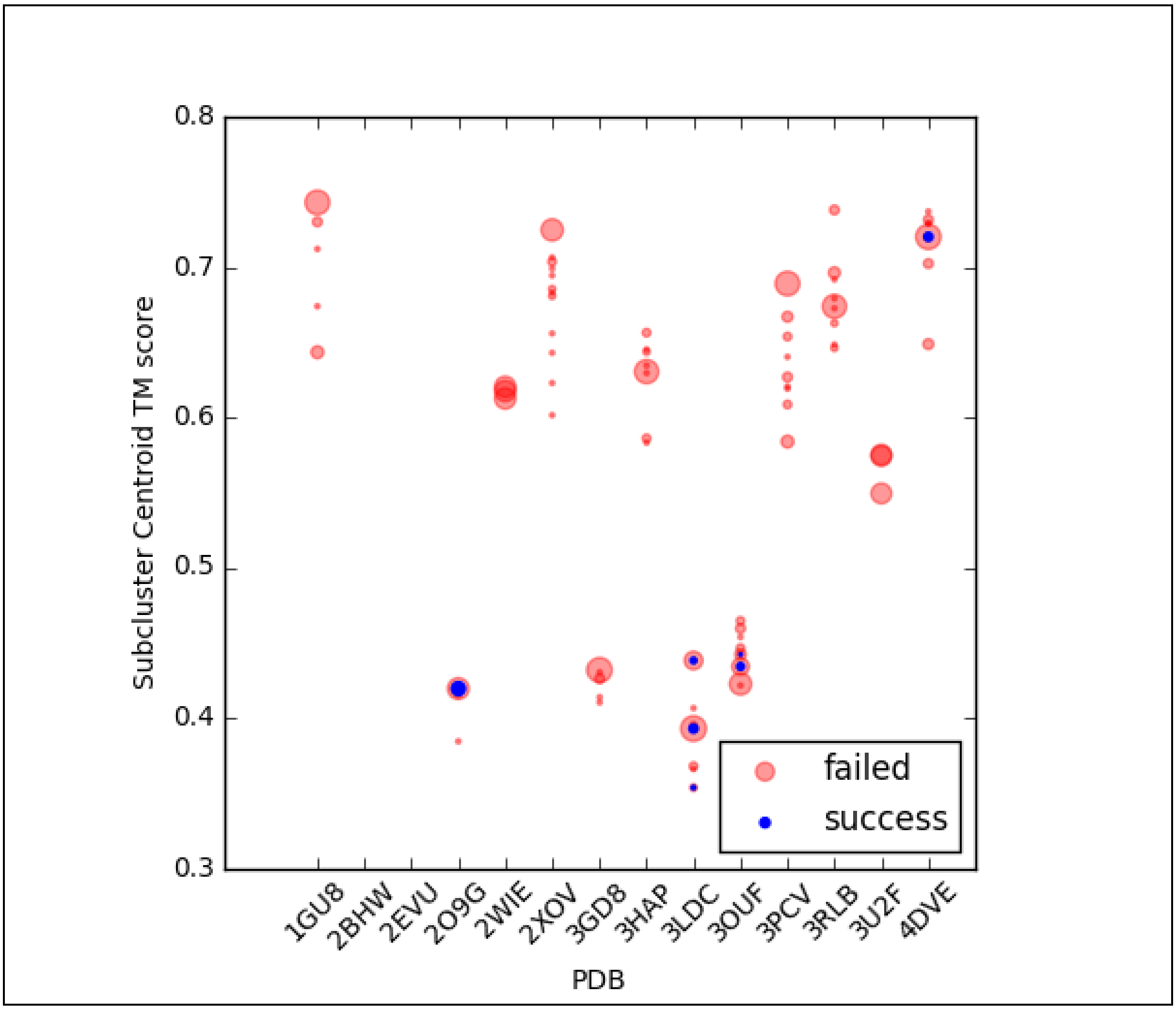

Figure S13 Scatterplot of TM score of the complete centroid model of the subcluster that was used to form the ensemble, against the target for METAPSICOV_S1 models. Points are sized by the number of ensembles and coloured red for failing ensembles and blue for successful ones.

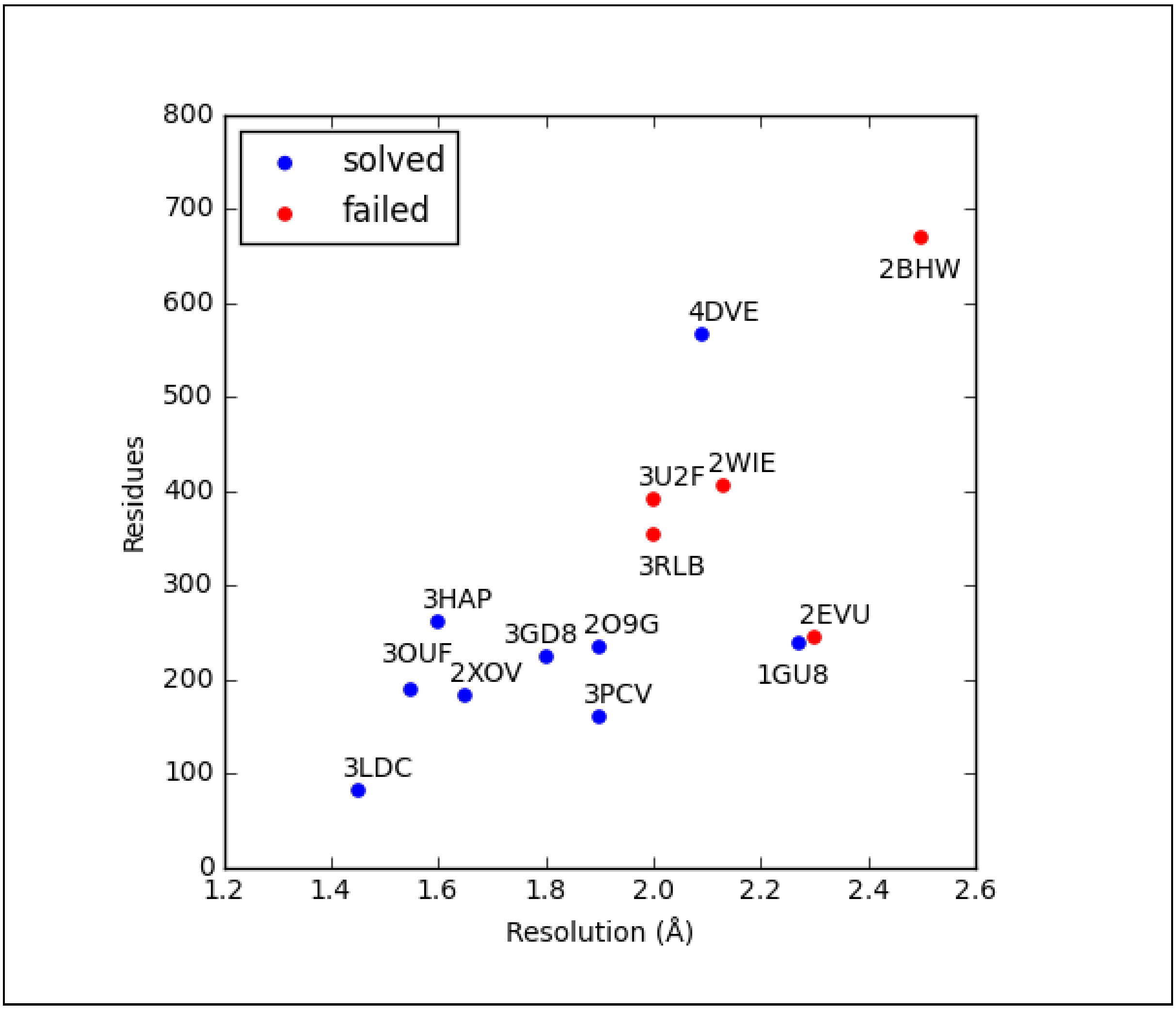

Figure S14 Results for attempting solution of TM proteins with all the different modelling protocols, mapped against target resolution and number of residues in the unit cell. Success are in blue, failures in red.

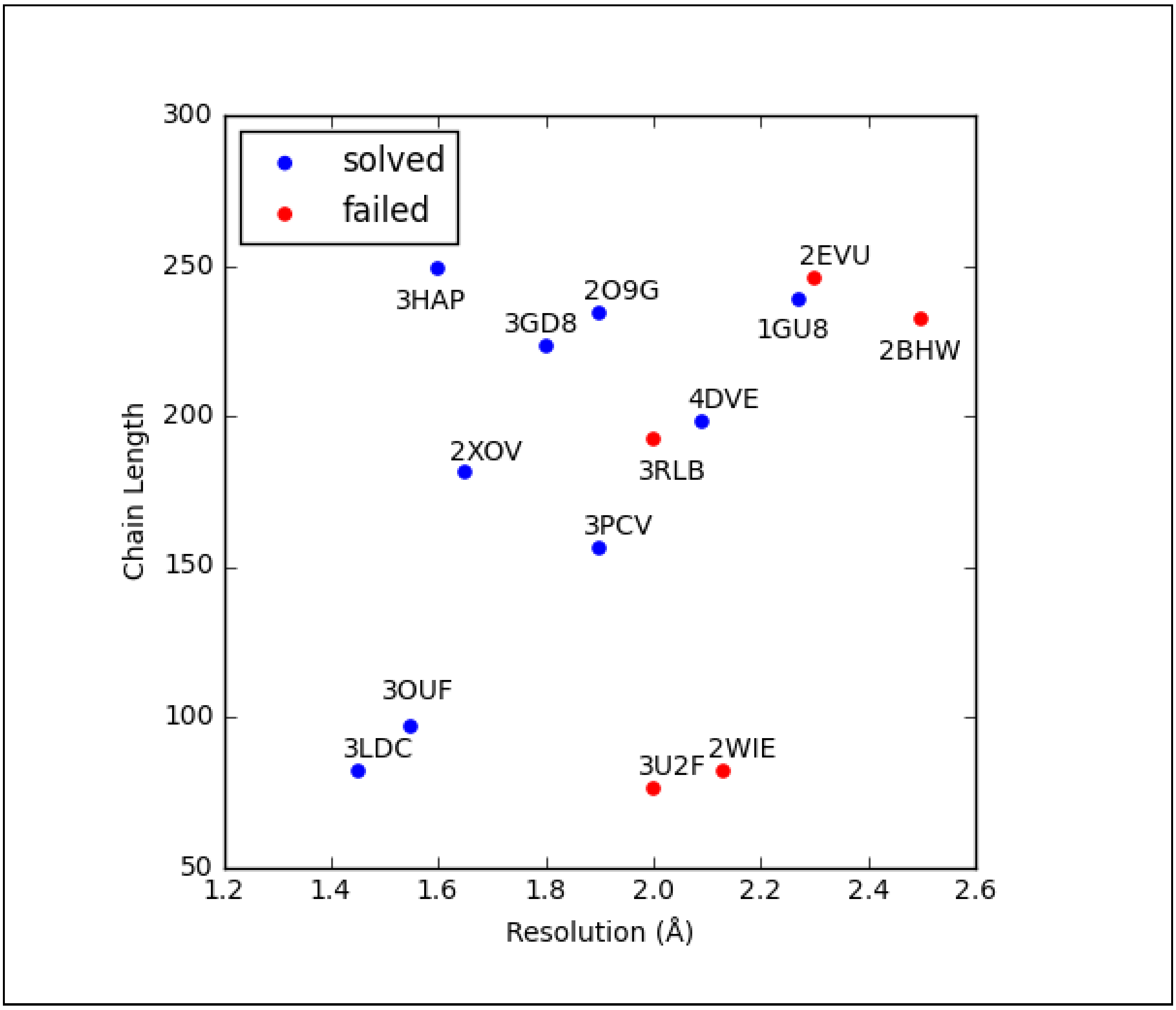

Figure S15 Results for attempting solution of TM proteins with all the different modelling protocols, mapped against target chain length and number of residues in the unit cell. Success are in blue, failures in red.

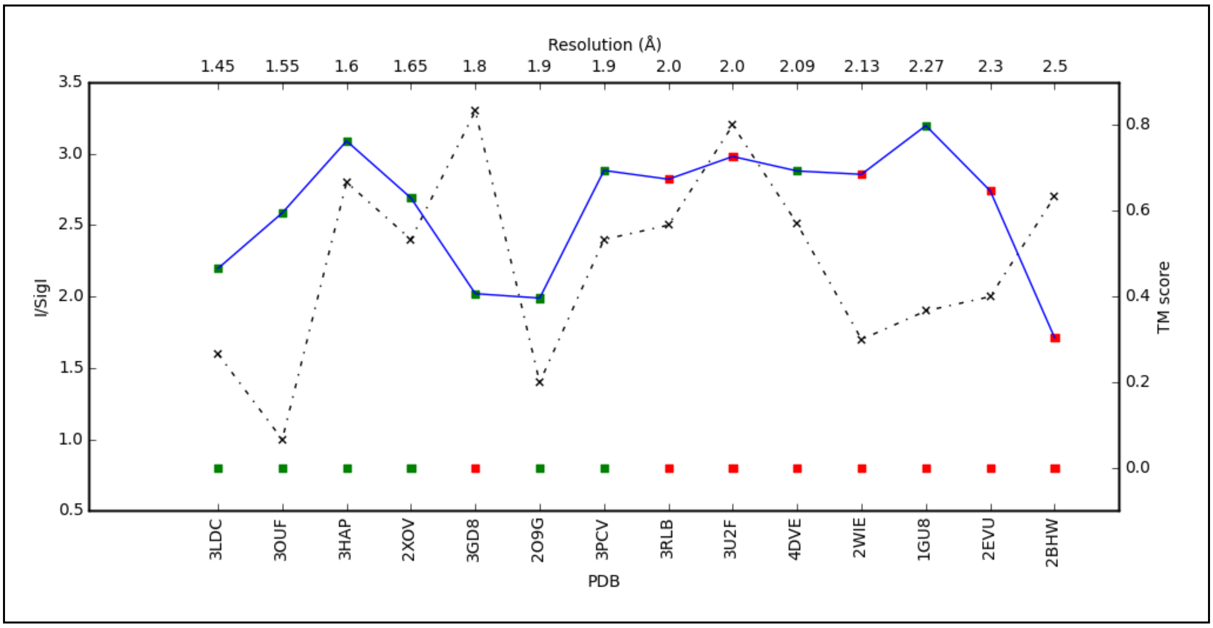

Figure S16 Plot of the maximum TM score for the best set of models (blue line), together with the I/SigI for the highest resolution shell (black dotted line), against the different targets ordered by resolution.The ideal helix solutions are plotted as squares along the bottom with a TM score of 0.0. Points are coloured green if the target could be solved red otherwise.

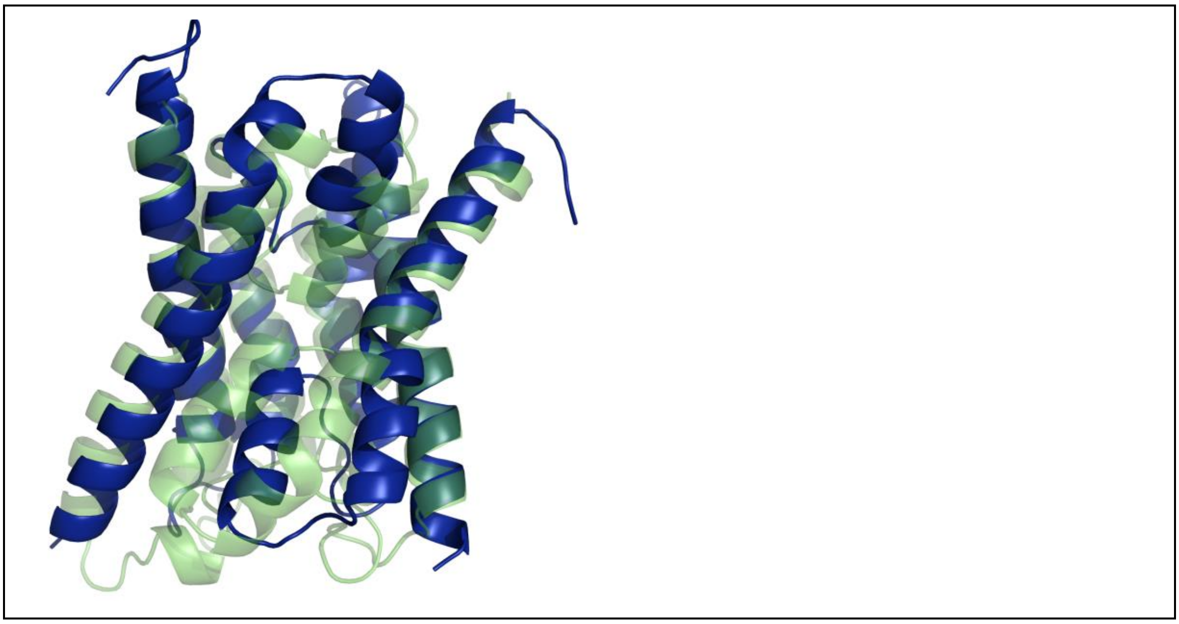

Figure S17 The two sections of the search model from ensemble c1_t95_r3_reliable (in blue) that overlap with the native structure of 2O9G and its symmetry mate overlaid on a single copy if 2O9G (in green) to show the overall match.

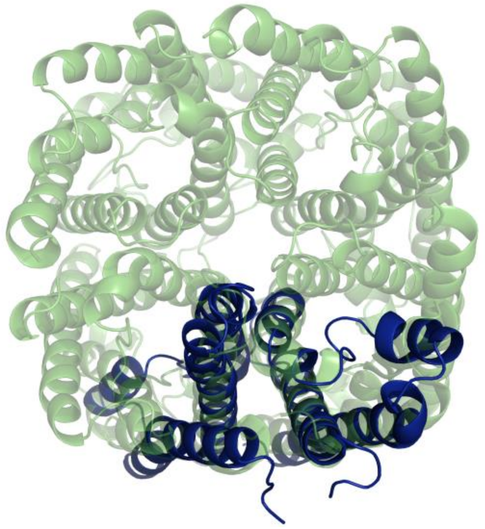

Figure S18 The biological assembly of 2O9G in green with the search model from ensemble c1_t95_r3_reliable in blue overlaid on it.

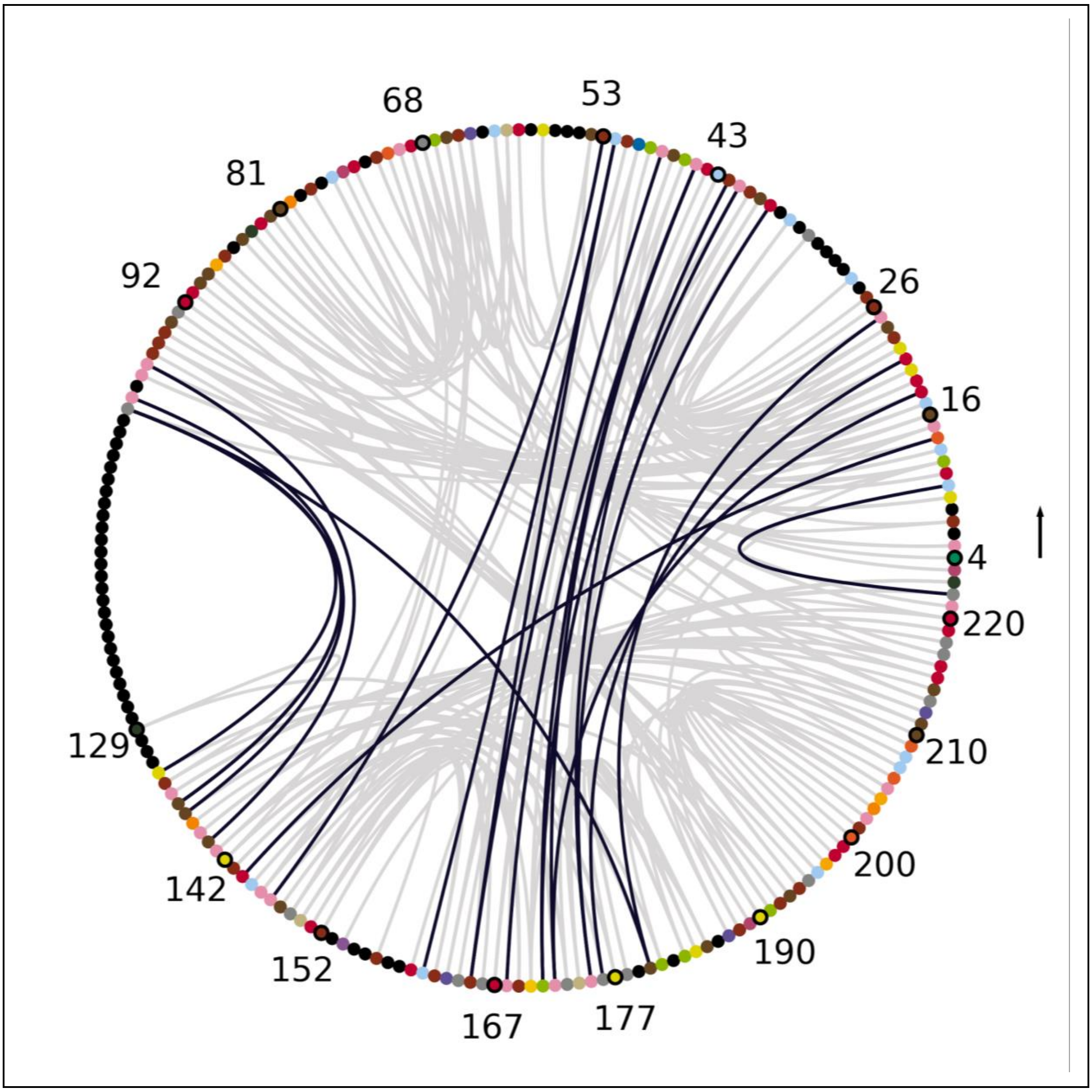

Figure S19 Top-L METAPSICOV_S1 contact predictions with correct interface contact pairs coloured in dark blue lines, and all others, both intra- and inter-molecular, in light gray. Reference contacts were extracted from the biological assembly of the X-ray crystal structure at 8Å distance between Cβ-Cβ (Cα in case of GLY) atoms.
